# Stimulated thyroid hormone synthesis machinery drives thyrocyte cell death independent of ER stress

**DOI:** 10.1172/JCI187044

**Published:** 2025-10-14

**Authors:** Crystal Young, Xiaohan Zhang, Xiaofan Wang, Aaron P. Kellogg, Kevin Pena, August Z. Cumming, Xiao-Hui Liao, Dennis Larkin, Hao Zhang, Emma Mastroianni, Helmut Grasberger, Samuel Refetoff, Peter Arvan

**Affiliations:** 1 Division of Metabolism, Endocrinology & Diabetes and; 2 Department of Molecular & Integrative Physiology, University of Michigan, Ann Arbor, Michigan, USA.; 3 Department of Medicine, The University of Chicago, Chicago, Illinois, USA.; 4 Department of Medicine, Division of Gastroenterology, University of Michigan, Ann Arbor, Michigan, USA.; 5 Department of Pediatrics and Committee on Genetics, The University of Chicago, Chicago, Illinois, USA.

**Keywords:** Endocrinology, Genetics, Apoptosis, Cell stress, Thyroid disease

## Abstract

It is now recognized that patient and animal models expressing genetically encoded misfolded mutant thyroglobulin (TG, the protein precursor for thyroid hormone synthesis) exhibit dramatic swelling of the endoplasmic reticulum (ER), with ER stress and cell death in thyrocytes — seen both in homozygotes (with severe hypothyroidism) and heterozygotes (with subclinical hypothyroidism). The thyrocyte death phenotype is exacerbated upon thyroidal stimulation (by thyrotropin [TSH]), as cell death is inhibited upon treatment with exogenous thyroxine. TSH stimulation might contribute to cytotoxicity by promoting ER stress or by an independent mechanism. Here we’ve engineered KO mice completely lacking *Tg* expression. Like other animals/patients with mutant TG, these animals rapidly developed severe goitrous hypothyroidism; however, thyroidal ER stress was exceedingly low — lower even than that seen in WT mice. Nevertheless, mice lacking TG exhibited abundant thyroid cell death, which depended upon renegade thyroidal iodination; cell death was completely suppressed in a genetic model lacking effective iodination or in *Tg-KO* mice treated with propylthiouracil (iodination inhibitor) or iodide deficiency. Thyrocytes in culture were killed not in the presence of H_2_O_2_ alone, but rather upon peroxidase-mediated iodination, with cell death blocked by propylthiouracil. Thus, in the thyroid gland bearing *Tg* mutation(s), TSH-stimulated iodination activity triggers thyroid cell death.

## Abstract 2

Thyroglobulin misfolding mutations trigger high endoplasmic reticulum (ER) stress and thyroid cell death. A thyroglobulin knockout mouse (no ER stress) reveals the cell death mechanism.

## Graphical abstract

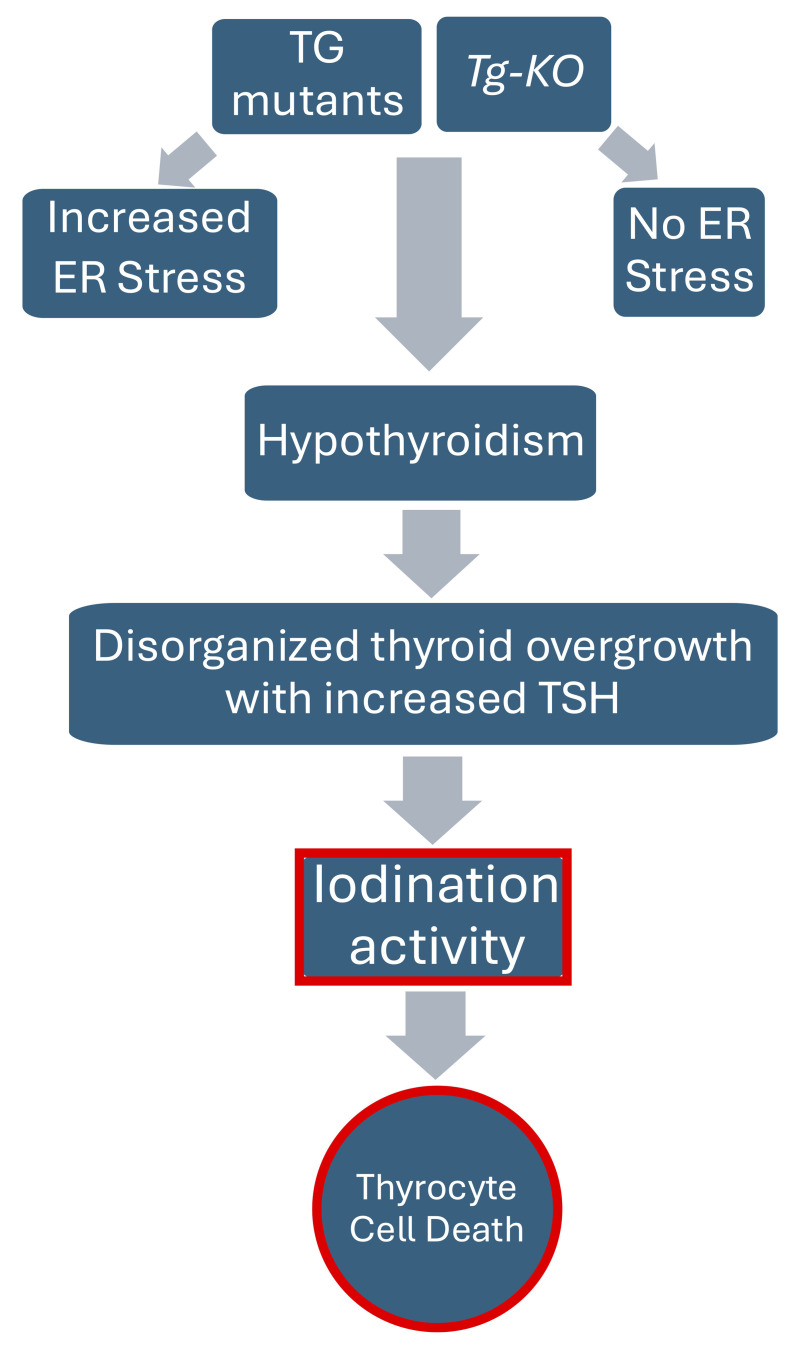

## Introduction

One of the well-recognized drivers of endoplasmic reticulum (ER) stress occurs in highly stimulated professional protein-secreting cell types, in which the ER folding environment is unable to keep up with unusually high levels of secretory protein synthesis (1). ER stress–mediated cytotoxicity has often been reported (2), sometimes attributed to an inadequate ER stress response, and other times attributed to an exuberant “terminal” ER stress response (3). ER stress is known to trigger adaptive responses such as increased ER folding machinery, ER protein degradation machinery (4–8), and ER-mediated cell death machinery (9–11). Coexisting ER stress and cell death has been particularly well documented in the thyroid gland (12–14), although ER stress has not been proved to be causative of thyroid cell death.

The thyroid gland uses a highly conserved mechanism for the biosynthesis of thyroxine (T_4_) that is found in all vertebrate organisms. Specifically, thyrocytes (the epithelial cells that synthesize thyroxine) form follicles in which a spherical epithelial monolayer encloses a central luminal cavity. This organization is designed for iodide uptake from the bloodstream with transepithelial transport to the apical lumen, accompanied by apical generation of H_2_O_2_, which provides the substrates needed for peroxidase-mediated iodination of apical proteins, primarily, thyroglobulin (TG, encoded by the *Tg* gene; refs. 15, 16). TG is apically secreted in vast quantity by thyrocytes, and its extracellular iodination within the follicle lumen (17) triggers the formation of T_4_ within TG itself (18–22), i.e., a postsecretory posttranslational modification (23, 24). When the thyroid gland is acted upon by thyrotopin (also known as thyroid-stimulating hormone [TSH]), multiple thyroidal events are stimulated, including thyrocyte proliferation, iodide uptake and iodination, and protein internalization from the extracellular apical lumen to the endolysosomal system of the surrounding thyroid follicular epithelial cells (25), which normally leads to proteolytic digestion of iodinated Tg and liberation of thyroid hormones for delivery to the bloodstream (26). TSH-mediated upregulation of these events represents the main physiological response to combat primary hypothyroidism (27).

TG is an unusually large, multidomain glycoprotein. At physiological levels of expression, large secretory proteins generate physiological ER stress, in part because of the increased challenges of folding, processing, and trafficking and the increased energy requirements that accompany those steps (28). Moreover, there are a large number of pathogenic variants in the *Tg* gene causing congenital hypothyroidism resulting from a defective TG product (29) that misfolds (14, 16, 30). Most of these structurally defective forms of TG (in humans and animal models) are subject to ER quality control, which means they are not allowed to travel forward from the ER into the more distal portions of the secretory pathway (30). Hypothyroidism from biallelic *Tg* mutation is a relatively uncommon condition that leads to a roughly 1,000-fold serum TSH elevation and massive ER stress with remarkable thyrocyte swelling (that can be attributed almost entirely to expansion of the ER itself), and we have reported that this condition is tightly associated with thyroid epithelial cell death (12–14). Thyroid cell death may contribute to the result that some patients with biallelic *Tg* mutations (as well as some animal models, such as the *rdw/rdw* rat) do not develop goiter (13). Interestingly, the far more common heterozygous condition results in only an approximately 10-fold elevation of serum TSH sufficient to stimulate normal circulating thyroid hormone levels, and, in this condition, abundant thyroid epithelial cell death is also observed (31).

Interestingly, this manner of thyroid cell death is almost completely abolished upon treatment of animals with exogenous thyroxine, which suppresses circulating TSH levels, which suggests the curious possibility that TSH stimulation might contribute not only to thyroid gland growth, but also to the phenotype of thyroid cell death (31). Based on considerations described in the paragraphs above, we and others have been operating under the assumption that TSH-mediated stimulation of thyrocyte death is driven by unremitting ER stress derived from misfolded Tg. In this study, we have set out to test this hypothesis, leading to a potentially entirely distinct and unexpected conclusion.

## Results

### Generation of Tg-KO mice.

We designed mice bearing deletion of *Tg* expression by homologous recombination (Supplemental Figure 1A; supplemental material available online with this article; https://doi.org/10.1172/JCI187044DS1), which resulted in replacement of 4.9 kB of the endogenous murine *Tg* gene, including removal of approximately 3 kB of *Tg* promoter plus 1.9 kB that includes *Tg* exons 1–3 (Supplemental Figure 1B). After selection of more than 100 ES colonies, we identified a suitable clone (Supplemental Figure 1, C and D). Mice generated from this clone yielded heterozygous animals that transmitted the KO allele through the germline and were then bred to homozygosity (Supplemental Figure 1E). RNA reverse-transcribed from the thyroid glands of homozygous mice yielded no detectable *Tg* mRNA, as measured with 11 distinct qPCR primer pairs spanning from exon 5 to exon 47 (Supplemental Figure 1F); similarly, thyroid tissue from these mice yielded no detectable TG protein by immunoblotting (Supplemental Figure 1G). Altogether, our data confirm complete absence of TG in homozygous KO mice.

**Figure 1:**
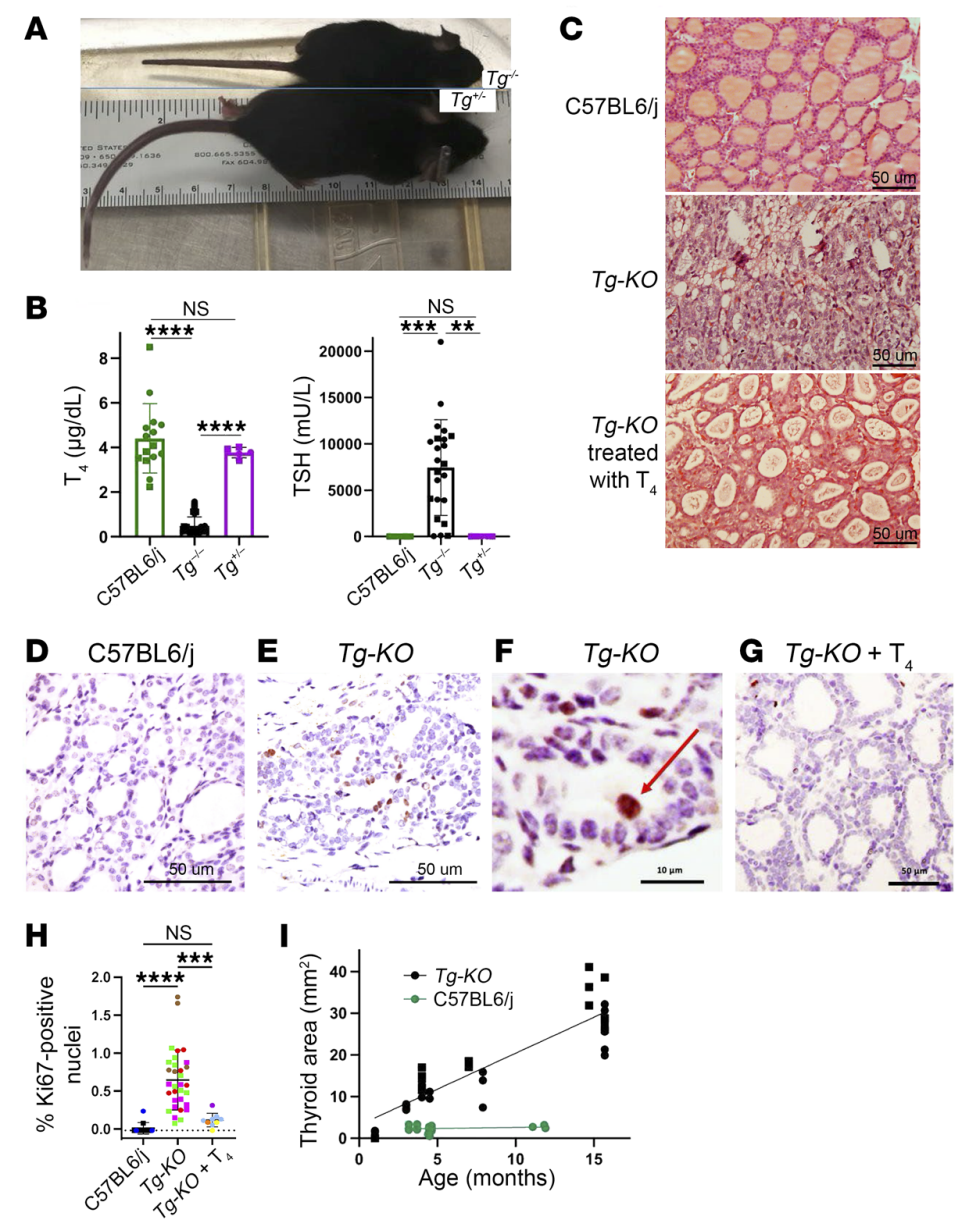
Phenotype of *Tg-KO* mice. (**A**) Whereas *Tg*^+/–^ mice (bottom) grow like WT mice (not shown in photo), *Tg*^–/–^ mice (top) are dwarfs. (**B**) The levels of serum total T_4_ and TSH in WT C57BL6/j (also referred to as *TG*^+/+^), *Tg-KO* (*TG*^–/–^), and *Tg^+/–^* heterozygous mice (*n* = 5–25 per group, 3.3 ± 1.3 mo; mean ± SD; **P < 0.01, ***P < 0.001, ****P < 0.0001; 1-way ANOVA with Tukey’s post hoc test). Each dot represents an individual animal (males, squares; females, circles). (**C**) Thyroid gland histology of *Tg*^+/+^ and *Tg-KO* mice without or with 6 weeks of exogenous T_4_ treatment beginning at the time of weaning (scale bar: 50 μm). (**D**–**G**) Ki67 immunohistochemistry of thyroid sections from *Tg*^+/+^ and *Tg-KO* mice without or with 6 weeks of exogenous T_4_ treatment. Scale bar: 50 μm (**D**, **E**, and **G**); 10 μm (**F**). (**H**) Quantitation of Ki67-positive nuclei as a percentage of total nuclei in thyroid images from *Tg*^+/+^ and *Tg-KO* mice without or with 6 weeks of exogenous T_4_ treatment (mean ± SD, ***P < 0.001, ****P < 0.0001 by 1-way ANOVA with Tukey’s post hoc test). Each color represents a different animal; each point is an independent thyroid section image (males, squares; females, circles). (**I**) Thyroid gland size as a function of age (males, squares; females, circles; the linear regression combines animals of both sexes).

Homozygous *Tg-KO* mice exhibited dwarfism (Figure 1A), as observed in other congenital models of hypothyroidism. Indeed, *Tg-KO* mice exhibited primary hypothyroidism with low circulating thyroxine levels and elevation of serum TSH to >5,000 mU/L (see Figure 1B — note that to improve fertility, homozygous pregnant mothers are treated with thyroxine, and this is continued in the presence of suckling pups in order to improve pup survival; the suckling pups may receive some thyroxine from breast milk, which may affect TSH levels in animals shortly after weaning).

### The thyroid glands of Tg-KO and heterozygous mice.

Histologically, in addition to perturbation of the normally homogeneous eosinophilic staining of the thyroid follicle lumen, the thyroid glands of homozygous *Tg-KO* mice revealed smaller and misshapen follicles with an obvious increase in thyroid tissue cellularity — both of which tended to be remediated in animals treated with thyroxine (Figure 1C). Elevation of serum TSH in primary hypothyroidism drives thyroid cell proliferation (32, 33), including thyrocyte growth into the follicle lumen, described as (noncancerous) papillae (34). Indeed, in the thyroid tissue of postweaning *Tg-KO* mice, Ki67 immunostaining was markedly increased (Figure 1E vs. Figure 1D; quantified in Figure 1H). In *Tg-KO* weanlings, some proliferating cells could be seen to grow into the thyroid follicular lumen (Figure 1F); thyrocyte overgrowth was prevented in postweaning *Tg-KO* mice that were intentionally treated with thyroxine (Figure 1, G and H), and inducible reexpansion of such cells could be observed in adult animals during the weeks following reversal of TSH suppression by withdrawal of exogenous thyroxine treatment (Supplemental Figure 2). Already detectable in weanlings, the abnormal appearance of occasional cells in the thyroid follicle lumen could be observed both by DAPI staining as well as residual Pax8 protein expression (Supplemental Figure 3A). Thus, in untreated *Tg-KO* pups immediately upon weaning from a thyroxine-treated mother or in adult animals following withdrawal of thyroxine supplementation, thyrocyte overgrowth occurs, including entry of these cells into the lumen of thyroid follicles. Accompanying this thyrocyte proliferation was progressive goiter development (roughly 5-fold enlargement of the thyroid gland at 4 months of age with a continued increase thereafter, Figure 1I and Supplemental Figure 1H).

**Figure 2:**
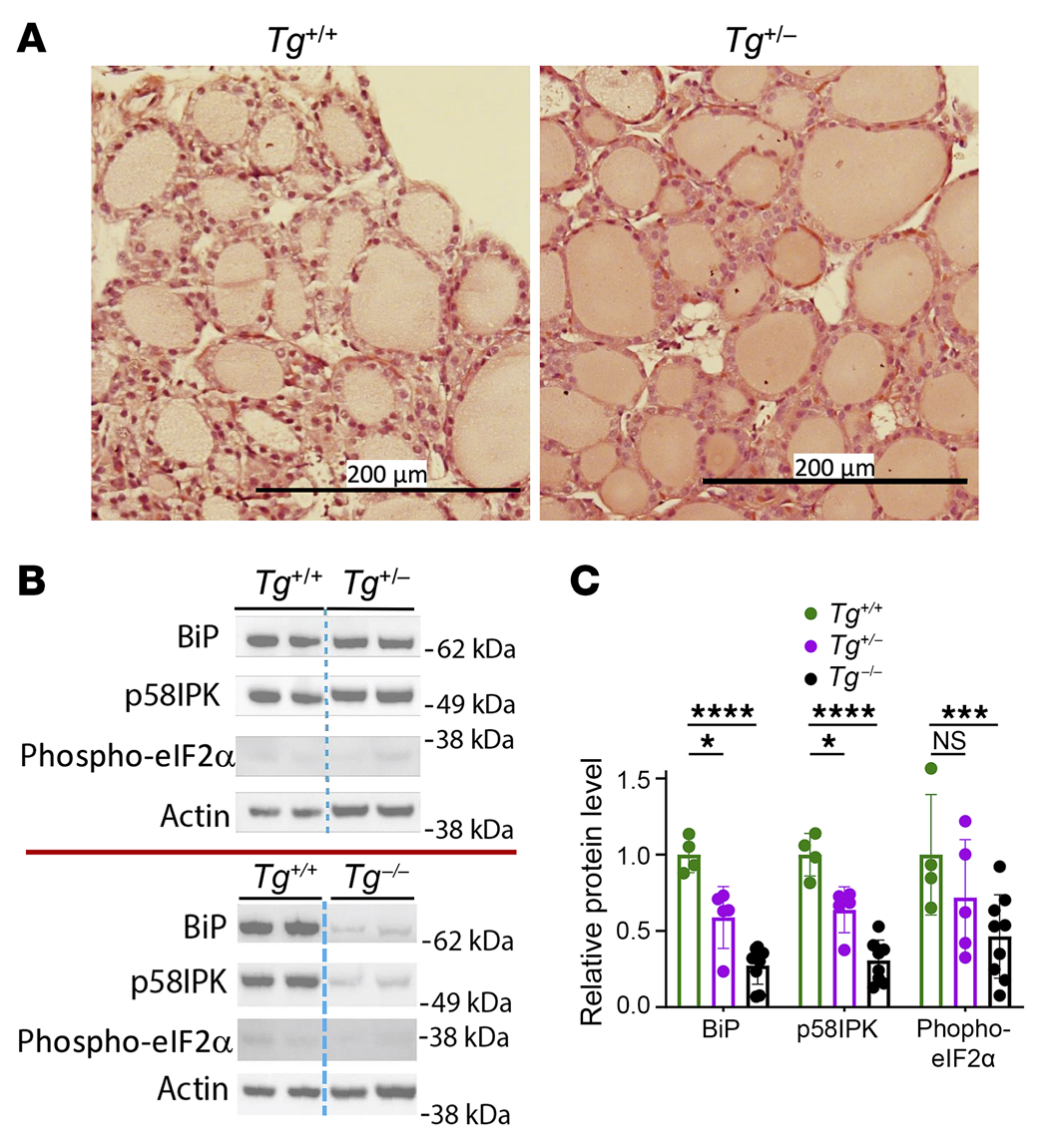
In the absence of TG expression, despite hypothyroidism, thyroid tissue exhibits unusually low biochemical levels of ER stress markers. (**A**) Thyroid gland histology of *Tg*^+/+^ control and *Tg*^+/–^ heterozygous mice at average age of 6.1 months (*n* = 2–3 per group; scale bar: 200 μm). (**B**) Immunoblotting of BiP, p58IPK, and phospho-eIf2α in the thyroid glands of *Tg*^+/+^ and *Tg^+/–^* heterozygotes (top) as well as *Tg*^+/+^ and *Tg*^–/–^ homozygotes (bottom; each panel comes from its own single gel/blot with intervening lanes removed). Actin is a loading control. (**C**) Quantitation of BiP, p58IPK, and phospho-eIf2α per unit actin, with heterozygotes and homozygotes normalized to WT C57BL6/j controls (*n* = 4–9 per group, mean ± SD. *P < 0.05, ***P < 0.001, ****P < 0.0001 (1-way ANOVA with Tukey’s post hoc test).

In *TG*^+/–^ heterozygotes, animals grew to normal size (represented in Figure 1A); thyroid histology appeared normal (Figure 2A); and unlike the elevated average TSH value of *Tg-KO* mice (in the thousands of mU/L), circulating TSH in *Tg*^+/–^ heterozygotes was ≤10 mU/L with normal circulating T_4_ levels (Figure 1B). Additionally, *Tg*^+/–^ heterozygotes exhibited a small decrease in the thyroidal levels of the ER chaperone BiP, cochaperone p5^8ipk^, or phosphorylated-eIF2α (Figure 2B, top; quantified in Figure 2C). In contrast, in *Tg-KO* mice, these ER stress markers were more markedly decreased (Figure 2B, bottom; quantified in Figure 2C), and this was also apparent upon immunofluorescence examination of BiP (Supplemental Figure 3B).

**Figure 3:**
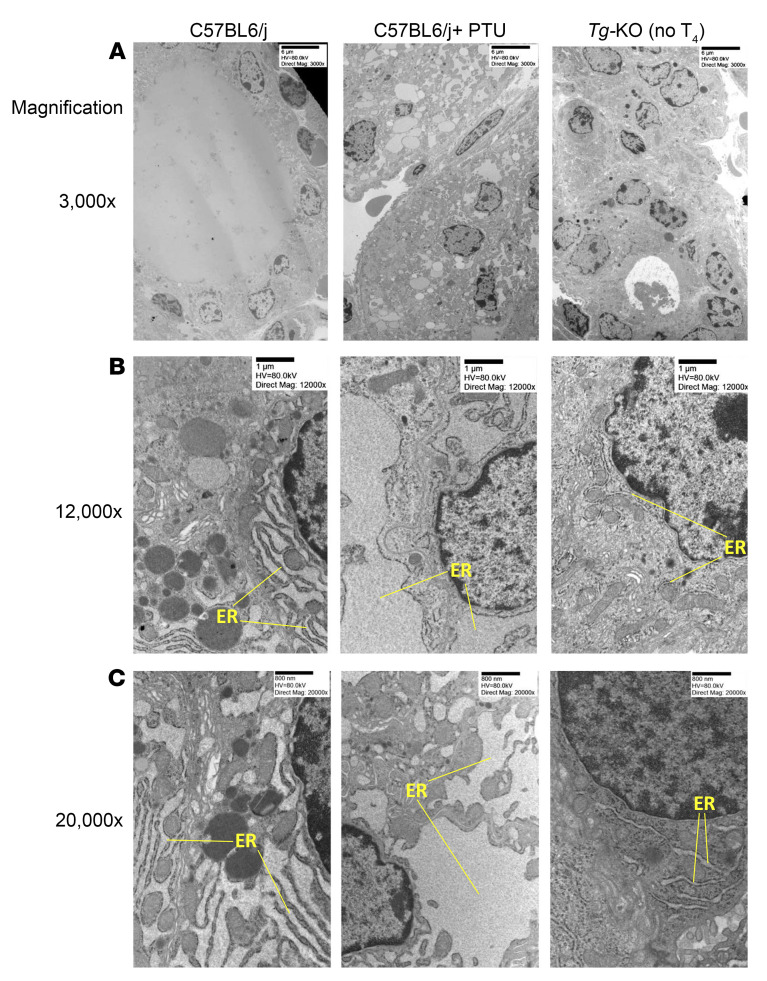
In the absence of TG expression, despite hypothyroidism, thyroid tissue exhibits an unusually small ER compartment, whereas hypothyroid animals that synthesize TG exhibit an expanded ER. Transmission electron microscopy of WT C57BL6/j mice untreated (2.3 months old, left column) or mice rendered hypothyroid with PTU treatment for 9 weeks (middle column) or *Tg-KO* mice rendered hypothyroid by removal of exogenous thyroxine treatment for 9 weeks (right column; average age 3 months; 2 animals per group). Image magnification and size bars are indicated. Original magnification, ×3,000 (**A**); ×12,000 (**B**); ×20,000 (**C**). The endoplasmic reticulum (ER) is identified.

By transmission electron microscopy, we contrasted the thyroidal response to hypothyroidism in *Tg-KO* mice with that of WT mice treated with the peroxidase inhibitor propylthiouracil (PTU, which blocks thyroidal iodination). As expected, untreated WT mice formed spherical thyroid follicles with a central protein-filled luminal cavity, but this pattern appeared disturbed in both *Tg-KO* and WT mice treated with PTU (Figure 3A). When viewed at higher levels of magnification, the ER of WT thyrocytes was easily recognizable at normal levels of bioactivity; the PTU-treated hypothyroid animals exhibited dramatic ER swelling, whereas the ER in *Tg-KO* hypothyroid mice was far more limited in size (Figure 3B). At the highest magnification employed, the thyrocyte ER in *Tg-KO* mice clearly exhibited a shrunken diameter in comparison to that observed even in untreated WT thyroid tissue (Figure 3C). The foregoing data support that the expression of endogenous TG is a major contributor to physiological ER stress in normal thyrocytes (35) and further establish that the thyroidal ER stress response to hypothyroidism is greatly dependent upon Tg protein expression.

### Thyroid cell death in Tg-KO mice occurs in the absence of ER stress.

**Figure 4:**
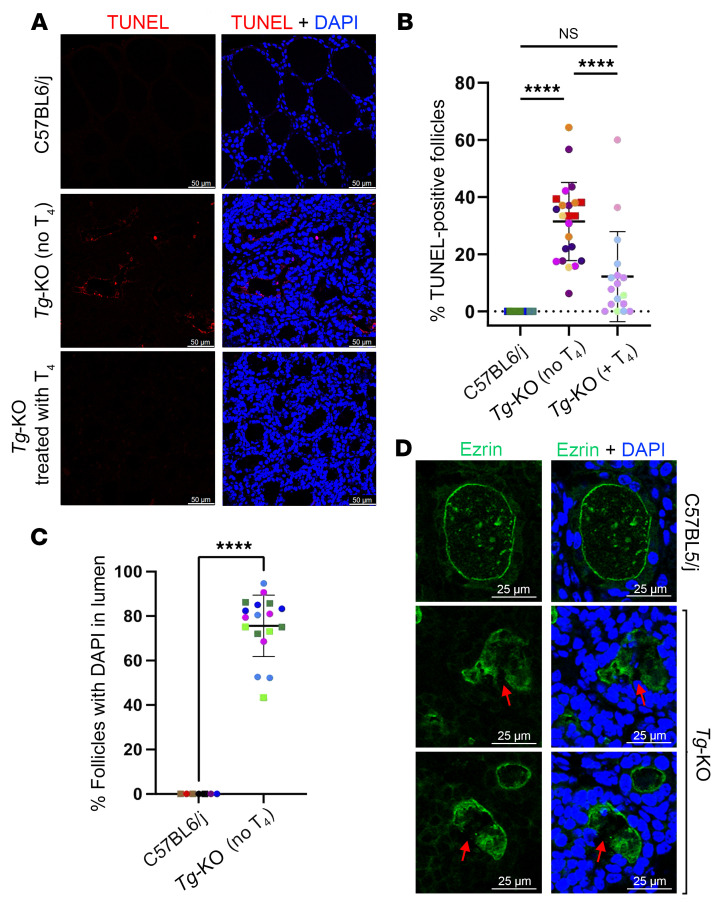
Thyroid cell death and discontinuity of the apical barrier in hypothyroid *Tg-KO* mice. (**A**) TUNEL labeling (red, with DAPI blue counterstain) in the thyroid sections of WT C57BL6/j and *Tg-KO* mice without or with 6 weeks of exogenous T_4_ treatment from the time of weaning (*n* = 4–6 per group beginning treatment at 1.2 ± 0.5 mo; scale bar: 50 μm). (**B**) Quantitation of TUNEL-positive follicles as a percentage of total follicles in each thyroid section image from the treatment conditions of **A**. Each color represents a single animal; each point is an independent image (males, squares; females, circles; mean ± SD, ****P < 0.0001; 1-way ANOVA with Tukey’s post hoc test). (**C**) Quantitation of follicles containing 1 or more DAPI-stained nuclear profile aberrantly located within the thyroid follicle lumen, as a percentage of total follicles in each thyroid section image from *Tg*^+/+^ and *Tg*^–/–^ mice (*n* = 5 per group, 1.6 ± 0.9 mo). Each color represents a different animal; each point is an independent image (males, squares; females, circles; mean ± SD, ****P < 0.0001; unpaired 2-tailed Student’s *t* test). (**D**) Immunofluorescence of ezrin (green, with DAPI blue counterstain) on the apical follicular luminal border in *Tg*^+/+^ and *Tg-KO* mice (*n* = 2–5 per group, 1.6 ± 0.9 mo; scale bar: 25 μm). Red arrows highlight apical discontinuity in ezrin immunostaining.

Both hypothyroid human patients with misfolded mutant TG and animal models (14, 30) exhibit evidence of dramatic thyroidal ER stress — and recent studies reveal that this (hypothyroidism with ER stress) is accompanied by thyroid cell death (12, 36–38). However, these findings do not prove that the observed thyroid cell death requires chronic unremitting ER stress as its trigger. An alternative explanation to be excluded could be that in the setting of primary hypothyroidism caused by a failure of secreted TG (despite active thyroid hormone synthesis machinery, ref. 12, including NADPH oxidase-mediated H_2_O_2_ generation, refs. 39, 40, and thyroid peroxidase-mediated iodination, ref. 17), thyroid cell death might emerge as a secondary consequence of these potentially cytotoxic TSH-stimulated enzymatic activities, as has been previously suggested (41). In support of this alternative, WT mice that exhibited physiological levels of thyroidal ER stress that were greater than those of *Tg-KO* mice, exhibited no detectable thyroid cell death by TUNEL staining (Figure 4A, top; quantified in 4B). Nevertheless, in *Tg-KO* weanlings we already observed ongoing thyroid cell death (Supplemental Figure 4A and Figure 4A, middle; quantified in Figure 4B). Moreover, whereas nuclear material was never seen in the thyroid follicle lumen of normal animals, *Tg-KO* mice exhibited abnormal, positive staining for DAPI in the lumen of most thyroid follicles (Figure 4C). Euthyroid *Tg*^+/–^ heterozygotes exhibited little thyroid cell death (Supplemental Figure 4B), and in *Tg-KO* homozygotes treated with thyroxine to suppress circulating TSH, thyroid cell death was similarly inhibited (Figure 4A, bottom; quantified in 4B). Moreover, within 5 days of withdrawal of exogenous thyroxine treatment, circulating TSH rose markedly and thyroid cell death resumed thereafter (Supplemental Figure 5, A–C). These data strongly suggest that, independent of ER stress, TSH stimulation in *Tg-KO* mice contributes to the thyroid cell death phenotype.

### Co-opting hormone synthesis machinery to drive thyroid cell death.

Enzymatically generated reactive metabolites in the thyroid follicle lumen (such as DUOX2-generated luminal H_2_O_2_, ref. 42, as well as thyroid peroxidase-generated oxidized iodide, ref. 41) are potentially cytotoxic — and dysregulation of the thyroid gland can lead to toxic iodination of thyroid lipids and proteins and can promote cell death (43, 44). It is said that, in the thyroid gland, the first line of protection from such damage lies within the structure of the follicle itself, by providing abundant TG enclosed with these toxic constituents within the follicle lumen, surrounded by an apical plasma membrane that is relatively impermeant to these metabolites and sealed between the epithelial cells by tight junctions (45). Normally, ezrin localization can be used to define the apical boundary of thyroid follicles (46–48) (Figure 4D, top); however, in the hyperstimulated thyroid glands of *Tg-KO* mice we frequently observed a loss of contiguity of the ezrin-marked apical boundary (Figure 4D, red arrows in bottom panels). We reasoned that such disorganization might result in exposure of unprotected thyrocyte regions to toxic metabolites normally generated in the iodination environment.

**Figure 5:**
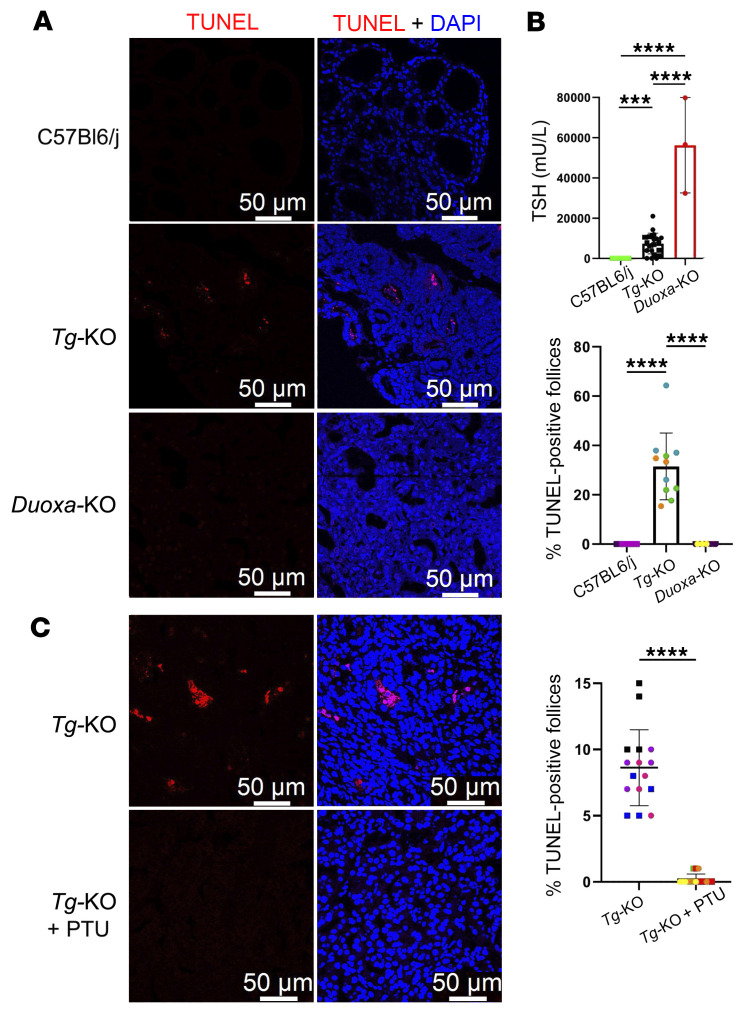
Thyroid cell death is inhibited by disruption upon impairment of the enzymatic machinery that leads to iodination. (**A**) Representative TUNEL labeling (red, with DAPI blue counterstain) in thyroid sections of WT C57BL6/j or *Tg-KO* mice without exogenous T_4_ treatment or *Duoxa-*KO mice (*n* = 3 per group, 1.2 ± 0.4 mo; scale bar: 50 μm). (**B**) Top*:* Serum TSH levels from the genotypes shown in **A** (*n* = 3–27 per group, 3.3 ± 1.4 mo. Mean ± SD; ***P < 0.001; ****P < 0.0001; 1-way ANOVA with Tukey’s post hoc test). Middle: Quantitation of TUNEL-positive follicles as a percentage of all follicles in each thyroid section image from the genotypes shown in **A**. Each color represents a different animal; each point is an independent image (males, squares; females, circles; mean ± SD; ****P < 0.0001; 1-way ANOVA with Tukey’s post hoc test). Bottom*:* Quantitation of TUNEL labeling shown in **C** using methodology of **B** (mean ± SD; ****P < 0.0001; unpaired 2-tailed Student’s *t* test). (**C**) TUNEL labeling (red, with DAPI blue counterstain) in thyroid sections of *Tg-KO* mice without or with 2 weeks of propylthiouracil (PTU) treatment (*n* = 4 per group beginning at 1.3 mo; scale bar: 50 μm).

Certainly, exogenous H_2_O_2_ added at supraphysiologic doses can kill thyrocytes (49), but in vivo, the contribution of DUOX2-generated H_2_O_2_ (42) to cytotoxicity in the thyroid gland might be explained either by a direct effect of H_2_O_2_ or by its supporting role as a cofactor in peroxidase-mediated iodination (44). To test this potential contribution of DUOX2-generated hydrogen peroxide, we compared TUNEL staining in the thyroid glands of *Tg-KO* mice to that observed in *Duoxa*-KO mice (in which part of *Duoxa2* and *Duoxa1* exons was deleted, resulting in thyroidal deficiency of DUOX2 and DUOX1, ref. 50). Remarkably, unlike the obvious thyroid cell death observed in *Tg-KO* mice (Figure 5A, middle; quantified in Figure 5B*,* middle), thyroid tissue of *Duoxa*-KO mice exhibited no thyroid follicles with positive TUNEL staining (Figure 5A, bottom; quantified in Figure 5B, middle). This cannot be explained by an insufficiently stimulated thyroid gland in *Duoxa*-KO mice, because circulating TSH levels in these mice were certainly not lower than those of *Tg-KO* mice (Figure 5B, top).

The presented data strongly suggest that DUOX2-generated H_2_O_2_ is an essential contributor to the thyrocyte death phenotype observed in iodination-competent thyroid tissue — but the data do not clarify whether the resulting TSH-stimulated levels of H_2_O_2_ generate direct toxicity or indirectly affect toxicity by providing this essential cofactor for peroxidase-mediated iodination (39). To test this, we treated *Tg-KO* mice for 2 weeks with PTU. Such treatment makes primary hypothyroidism worse but, even if thyroidal H_2_O_2_ levels were to be further increased by TSH stimulation, this cannot overcome the PTU inhibition of thyroid peroxidase (51). Remarkably, 2 weeks of PTU treatment almost completely eliminated all detectable thyroid cell death (Figure 5C, quantified in Figure 5B, bottom).

Of course, enzymatically generated H_2_O_2_ alone is potentially capable of generating a cytotoxic response in 293 cells (40), and supraphysiologic doses of H_2_O_2_ are capable of even killing thyrocytes (44). Using the Cytotox-Glo assay system (52), PCCL3 thyrocytes exposed to H_2_O_2_ generation by glucose + glucose oxidase (2.7 U/L) showed little or no evidence of cell death, although a vast (>160-fold) excess of glucose oxidase (442 U/L) was sufficient to kill all PCCL3 thyrocytes within 24 hours (Supplemental Figure 6) — and this cell death cannot be inhibited by simultaneous addition of PTU even at 1,000 μM (Supplemental Figure 6).

**Figure 6:**
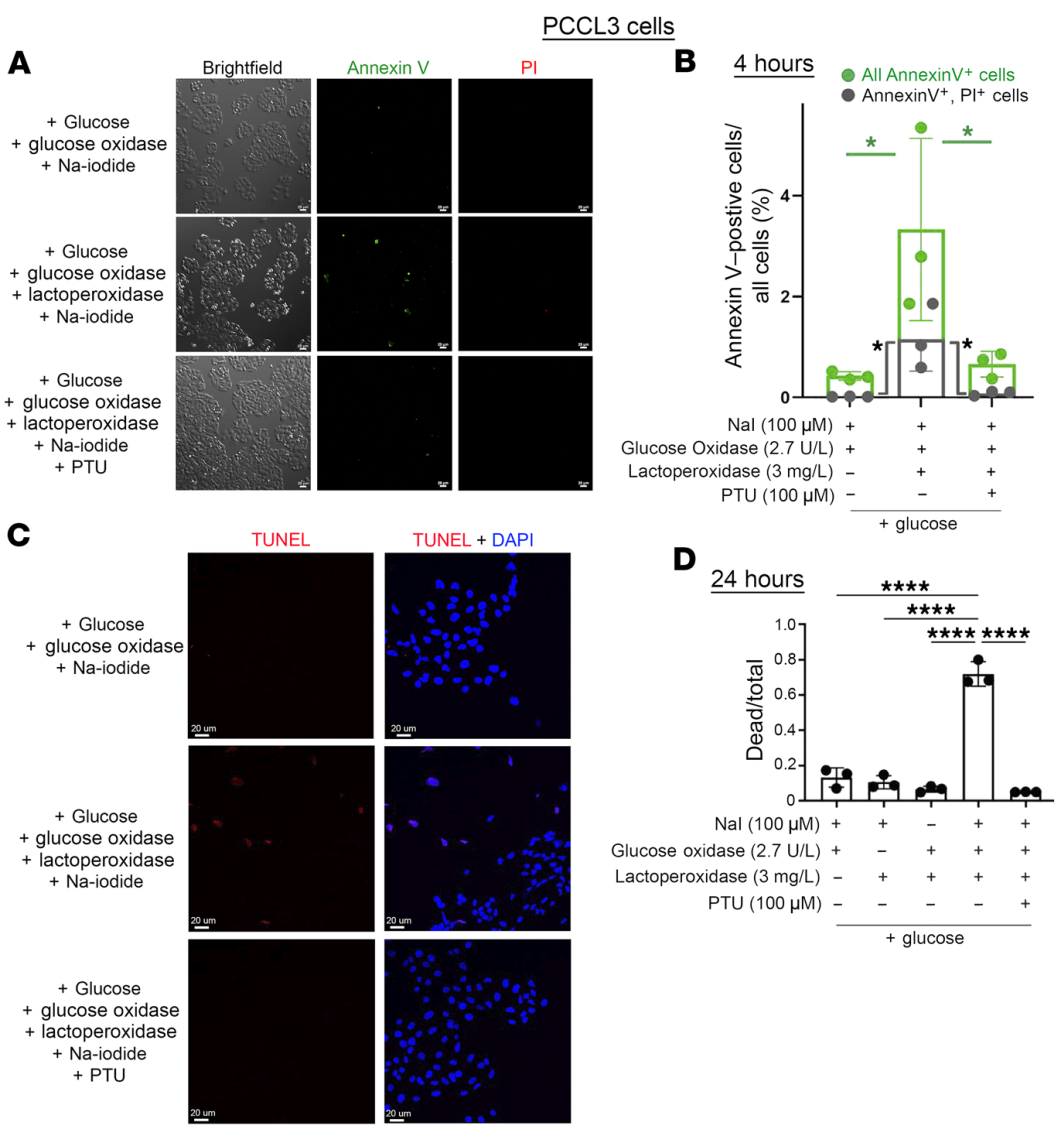
In vitro, moderate enzymatically generated extracellular H_2_O_2_ is not sufficient to trigger thyrocyte cell death but becomes cytotoxic when coupled to peroxidase-mediated iodination. PCCL3 cells growing in media containing 17.5 mM glucose were treated for different times under 3 distinct sets of conditions (except for time of treatment, these 3 distinct conditions were identical in all panels). (**A**) After a 4-hour exposure to the conditions described at left, the cells were labeled with Annexin V–FITC (green) and propidium iodide (PI, red). Scale bar: 25 μm. Identical fields were imaged under bright-field conditions (enabling counting of all cells) and epifluorescence; representative images are shown (**B**) Quantitation of Annexin V–positive PCCL3 thyrocytes that were concurrently positive for PI staining (dark gray symbols and bars) together with annexin V-positive cells that were negative for PI staining add up to all Annexin V–positive cells (additive green symbols and bars). The data were quantified as a fraction of all cells present from 3 independent replicate experiments (mean ± SD, *P < 0.05, 1-way ANOVA with Tukey’s post hoc test; the 3 conditions examined 30,406 cells, 22,836 cells, and 36,530 cells, respectively). (**C**) After an 8-hour exposure to the conditions described at left, TUNEL labeling (red, with DAPI counterstain in blue) was performed in 5 independent replicate experiments, with 5 independent fields examined for each condition in each experiment. From these 25 fields, in the presence of active lactoperoxidase, 30.6% of cells were TUNEL positive; when peroxidase was inhibited with PTU only 1.86% of cells were TUNEL positive (*P* < 0.001). Scale bar: 20 μm. (**D**) After a 24-hour exposure to the conditions indicated below the graph, the CytoTox-Glo cytotoxicity assay revealed significant cell death only in the presence of the complete iodination cocktail, inhibited by PTU (3 independent experiments, mean ± SD, ****P < 0.0001, 1-way ANOVA with Tukey’s post hoc test).

Similar to TSH-mediated thyrocyte overgrowth with discontinuities in the apical boundary surrounding the thyroid follicle lumen (Figure 4D), PCCL3 (or FRTL5) cells do not form a tight epithelial barrier comparable to that of the thyroid in vivo (53) (and these cells are typically grown at subconfluent density); thus, PCCL3 cells are susceptible to paracellular diffusion of small molecules. However, glucose oxidase at the lower 2.7 U/L dose in the presence of lactoperoxidase ([LPO], which is known to have similar capability to TPO in supporting iodination, ref. 54) plus 100 μM sodium iodide, when combined together, triggered the appearance of Annexin V positivity within 4 hours (Figure 6A, quantified in 6B); TUNEL positivity within 8 hours (Figure 6C); and 70% cell death at 24 hours by the Cytotox-Glo assay (which quantifies release of cytoplasmic proteases into the medium, Figure 6D). In each type of assay, thyrocyte cell death was blocked either by omitting one component of the iodination system or by addition of PTU (which inhibits LPO as it inhibits TPO, ref. 55) (Figure 6). Iodination activity was confirmed by performing the same assay for 4 hours in serum-free medium using PCCL3 cells bearing CRISPR-mediated *Tg-KO* (35, 56) in the presence of radioiodide tracer (Supplemental Figure 7, lanes 4+5). Thus, whereas no 2 of the 3-component iodination system was sufficient to trigger enhanced cytotoxicity, iodination conditions yielded a highly significant level of thyroid cell death that was essentially completely blocked by 100 μM PTU (Figure 6), which is a condition that blocked iodination (Supplemental Figure 7, lanes 1+2) and has been reported to block peroxidase-mediated death of human thyrocytes (41). Notably, surface-positive staining with Annexin V–FITC at 4 hours seemed to begin in cells whose nuclei did not stain positively with propidium iodide (Figure 6, A and B), indicating apoptosis (57) as the cause of cell death under iodination conditions.

**Figure 7:**
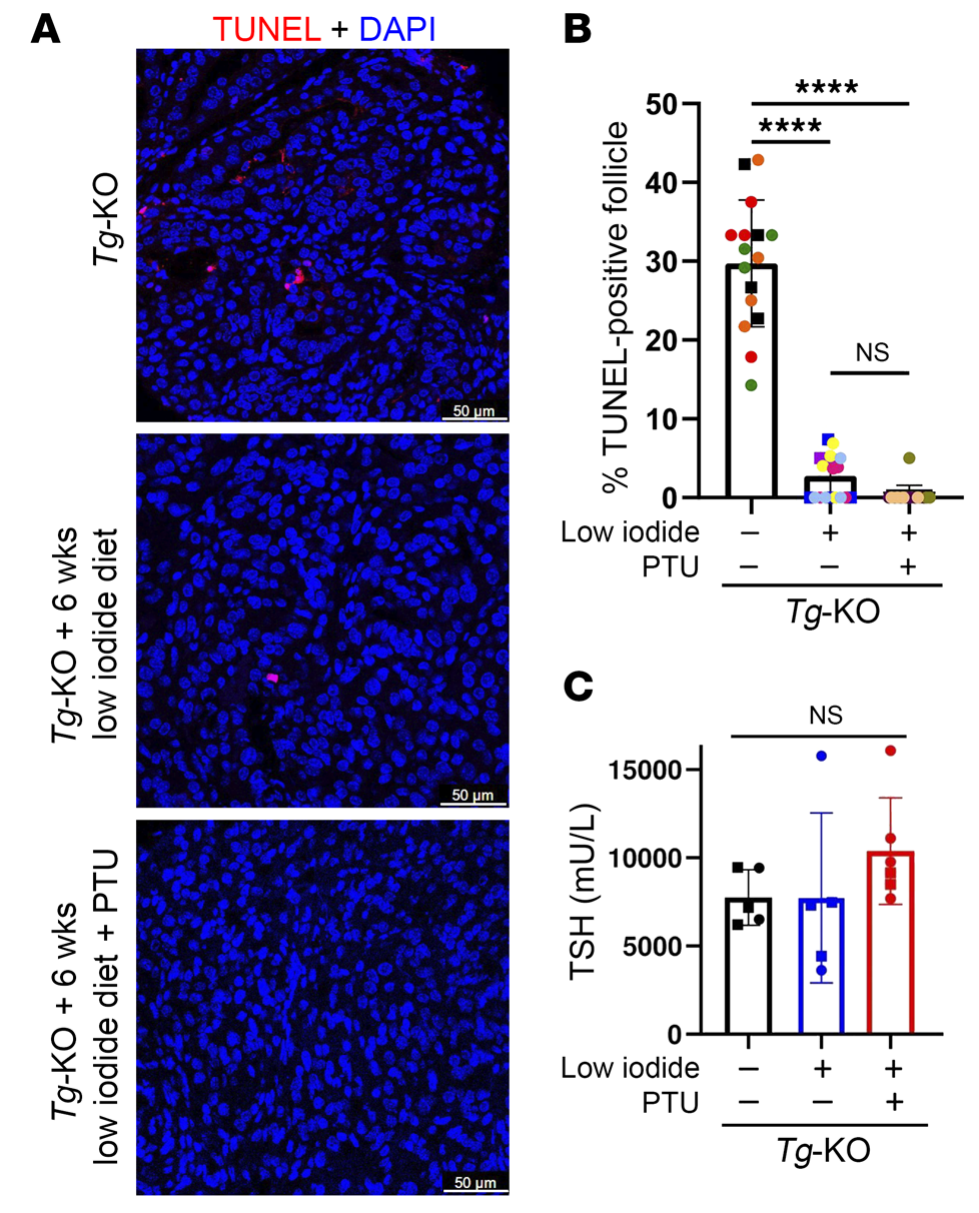
Thyroid cell death is suppressed in *Tg-KO* mice on a low-iodide diet. (**A**) Representative TUNEL labeling (red, with DAPI blue counterstain) in thyroid sections of *Tg-*KO mice fed normal chow, low-iodide diet for 6 weeks, or low-iodide diet containing PTU for 6 weeks (animals euthanized at age 2.5 ± 0.1 mo; *n* = 4–5 animals per group). Scale bar: 50 μm. (**B**) Quantitation of TUNEL-positive follicles in each thyroid section from the treatments shown (as in **A**). Each color represents a different animal; each point is an independent image (males, squares; females, circles; mean ± SD; ****P < 0.0001; 1-way ANOVA with Tukey’s post hoc test). (**C**) Circulating TSH levels from mice treated with the conditions shown in **A**. Each dot represents an individual animal (males, squares; females, circles).

Finally, because thyrocyte cell death is blocked either by lack of any element of the 3-component iodination system, we tested whether thyroid cell death in vivo might be limited in *Tg-KO* animals transitioned to a low-iodide diet for 6 weeks. Because of intrathyroidal iodide recycling by DEHAL1, one might expect that iodination-mediated thyroid cell death would continue unabated in animals on a low-iodide diet, which would be expected to develop even more severe hypothyroidism (58). However, limiting iodide availability in *Tg-KO* mice blocked TUNEL staining and additional treatment with PTU had little additional effect on circulating TSH or inhibition of thyroid cell death (Figure 7). Altogether, these data establish that without any enhancement of ER stress, as long as the machinery supporting iodination is enzymatically active (and there is incomplete integrity of the apical barrier, as may occur in states of thyroid overgrowth), the generation of oxidized iodide, linked to “renegade iodination,” kills thyrocytes in vivo and in vitro.

## Discussion

ER stress is a common feature in the pathology of numerous diseases, including neurodegeneration, cancer, diabetes/metabolic diseases, and inflammation; and it is recognized that ER stress can induce cell death by various mechanisms (59, 60). Recent studies have highlighted thyrocytes as a model of cell survival and/or death under conditions of perturbed ER proteostasis with ER stress (12, 35, 38). The thyroid is a particularly good model for such studies, as it is exceptionally easy to detect thyroid cell death in vivo, whereupon entry into the lumen of thyroid follicles, dead thyrocytes remain entrapped as they gradually disintegrate over time (12). Features that augment ER stress, such as deficiency of HRD1-mediated ER-associated protein degradation, exacerbate the problem under conditions of TG misfolding in the ER, with whole-follicle death and involution (31). Thus, it had tentatively been concluded that, in homozygous patients bearing mutations encoding misfolded mutant TG, which causes dramatic ER stress and stress response, that the proximal and primary cause of thyrocyte cell death is from ER stress (14).

What has been largely overlooked is the possibility that TSH stimulation of thyrocytes, which includes growth into the thyroid follicle lumen (13) that is seen in patients (12), could expose thyrocytes to a more cytotoxic environment triggering cell death (41). Although unexpected, here we found that overgrowth of thyrocytes in hypothyroid *Tg-KO* mice (Figure 1, E, F, H, and I, and Supplemental Figure 2) was linked to thyroid cell death (Figure 4, A–C, and Supplemental Figure 4A), even though they exhibited thyroidal ER stress levels that were not only lower than those of animals expressing misfolded TG caused by biallelic mutation, they actually exhibited ER stress levels lower than those of thyrocytes in normal WT mice (Figure 2, B and C, Figure 3, and Supplemental Figure 3B). This cell death is extremely likely to require TSH stimulation, as it was obviously suppressed in animals given exogenous thyroxine treatment (Figure 4A) and resumed after TSH levels rise upon thyroxine withdrawal (Supplemental Figure 5). We posit that several factors contribute to thyrocyte cell death under these conditions. First, generation of both H_2_O_2_ and oxidized iodide (from the combination of DUOX and TPO activities) is robust under conditions of TSH stimulation (61). Second, TG protein in mass quantity ordinarily presents itself as the iodination substrate in the follicle lumen (where it successfully competes for oxidized iodide, ref. 51) so that when TG is not available, luminal levels of oxidized iodide are likely to be higher than normal. Third, there are visible discontinuities in the apical membrane barrier in the thyroid tissue of *Tg-KO* mice (Figure 4D) in conjunction with thyrocyte overgrowth into the follicle lumen (Figure 1F, Supplemental Figure 2, and Supplemental Figure 3A). The current evidence supports that these features, together, are sufficient to trigger thyroid cell death in the absence of ER stress.

Although TSH stimulates thyroidal generation of H_2_O_2_, it is the virtually complete inhibition of cytoxicity by treatment of animals with PTU for 2 weeks (Figure 5) or feeding a low-iodide diet for 6 weeks (Figure 7) that convinced us that thyroid cell death in *Tg-KO* mice is not driven directly by H_2_O_2_ but by peroxidase-mediated formation of oxidized iodide for iodination. Nevertheless, H_2_O_2_ plays an essential supporting role as a cofactor for peroxidase-mediated iodination, which is why hypothyroid animals lacking DUOX activity were similarly protected from thyroid cell death (Figure 5). Moreover, under the in vitro conditions shown in Figure 6, PCCL3 thyrocytes in culture were killed only upon concurrent addition of all components of the system that together drive iodination (Supplemental Figure 7), and thyrocyte cell death was once again blocked upon chemical inhibition of peroxidase activity. The early detection of Annexin V–positive (propidium iodide-negative) thyrocytes in vitro (Figure 6, A and B) and activation of caspase-3 in vivo (Supplemental Figure 4A) suggest that cytotoxic iodination, which can include both proteins (Supplemental Figure 7) and lipids (41), triggers thyrocyte cell death via apoptosis (41, 62).

The foregoing findings notwithstanding, we cannot exclude that ER stress might possibly also contribute to thyroid cell death in patients and animal models expressing misfolded mutant TG. An interesting piece of evidence for consideration of this possibility is that heterozygous mice lacking one expressed allele of *Tg* exhibited very little thyroid cell death (Supplemental Figure 4B) whereas heterozygous mice bearing one WT *Tg* allele plus one allele encoding misfolded mutant TG protein exhibited increased thyroidal ER stress with thyroid cell death (31). Thus, one might argue that, in patients and animals with hypothyroidism caused by misfolded mutant TG, ER stress–mediated cell death and iodination-mediated death mechanisms might operate concurrently. However, it must be noted that heterozygotes lacking one expressed allele of *Tg* dis not show any elevation of circulating TSH (Figure 1B) whereas heterozygotes bearing one allele driving the expression of misfolded mutant TG exhibit subclinical hypothyroidism with elevated TSH (31). Thus, it remains quite possible that the main difference in cell death between the two heterozygous model systems might be the level of TSH stimulation rather than the level of ER stress. Indeed, thyroxine treatment of animals with heterozygous expression of misfolded mutant TG does not appreciably lower ER stress markers in the thyroid gland but completely inhibits thyroid cell death (31). This observation lends support to the notion that events downstream of TSH stimulation may be a more important factor than ER stress in the thyroid cell death phenotype observed in patients expressing misfolded mutant TG.

Finally, we emphasize that despite ongoing thyroid cell death in patients and animals with misfolded mutant TG, the TSH-driven proliferative response resulted most often in net growth of the thyroid gland (Figure 1I and Supplemental Figure 1H). Thus, it is the balance of thyroid cell growth and death that determines the clinical presentation and size of goiter (13).

## Methods

### Sex as a biological variant.

Our findings are expected to be relevant for both sexes; therefore, data from both males and females were analyzed and were included, with sex of each animal indicated in graphs. There were similar findings for both sexes.

### Mice.

All mice were in a C57BL6/j background. The *Tg-KO* allele was created by homologous recombination, as described in Results. Homozygous *Tg-KO* mice were generated either by crossing *Tg*^+/–^ heterozygous parents, or by crossing *Tg*^+/–^ heterozygous with *Tg-KO* homozygous parents, or by breeding homozygous *Tg-KO* parents while they receive T_4_ supplementation. When used, T_4_ treatment of *Tg-KO* mice was performed by supplementation of drinking water with T_4_ (1 μg/mL, T2501, MilliporeSigma). Both male and female animals were used. Unless otherwise indicated, mice were 1.0–2.8 months old. When used, mice were fed low-iodide chow containing PTU (1.5 g/kg, Envigo, TD.95125; normal content would require adding iodine at 0.7 mg/Kg from potassium iodate). When used, mice were fed a modified AIN-93M research diet formulated to be <10 μg iodine/100 g chow (Bio-Serve, F7853).

For the experiments in Supplemental Figure 5, *Tg-*KO mice began T_4_ supplementation (noted above) immediately after weaning for at least 15 days. Thereafter, at different times the T_4_ supplementation was withdrawn, in order to vary the withdrawal period from 1 to 15 days. All mice were ultimately euthanized at 30 days after weaning.

### Generation and genotyping of Tg-KO mice.

HindIII linearized, band-purified targeting vector DNA was electroporated into R1 (mouse) ES cells and individual clones plated into 96-well plates for positive selection of neomycin resistance and negative selection for TK-mediated incorporation of ganciclovir. Candidate KO clones were screened by conventional PCR with primer sets targeting both the 5′ homologous flanking sequence and within the disruption insert. Candidate clones were further confirmed by Southern blotting performed on nitrocellulose filters. 10 μg HindIII-digested mouse ES cell genomic DNA was resolved by 1% agarose gel electrophoresis. A 498 bp DIG labeled probe, which hybridizes 5′ upstream of the replacement vector, was generated by PCR using 5′ forward primer CTTACAGCATGGGCAGCAGACTC and 5′ reverse primer GTCTCCTCCACGGGGGTCAG and Roche labeling kit (catalog 11636090910). Confirmed positive ES clonal cells were used for 129X1/SVJ blastocyst injection by the University of Michigan Transgenic mouse core facility followed by implantation of blastocysts into pseudo pregnant 129X1/SVJ hosts. Tail biopsy DNA from resulting chimeric mice was analyzed by PCR for presence of recombination. Germline candidates were mated to C57BL6/j mice to confirm germline transmission and further backcrossed C57BL6/j. One resulting *Tg-KO* mouse line was established. The PCR primers used to identify the WT *Tg* allele by tail biopsy DNA were forward CAGGGCCCTTAAGCATGCCTGA and reverse ATAGCTCACAGGGGCGGAGTGG. Upon conventional PCR, the WT *Tg* allele generates an approximately 450 bp product. The KO allele was identified with forward Neo-forward primer GCCCCAGCTGGTTCTTTCCG and Neo-reverse primer GCGTTCCTTGCGCAGCTGTG generating an approximately 850 bp product.

### Primary antibodies.

We used rabbit anti-TG (Abcam, ab156008; Santa Cruz Biotechnology, 365997), mouse anti-Actin (Proteintech, 66009-1-Ig); rabbit anti-BiP was previously described (36); rabbit anti-ERdj6 (Cell Signaling Technology, 2940); rabbit anti-phospho-eIF2α (Cell Signaling Technology, 9721); mouse anti-eIF2α (Cell Signaling Technology, 2103), rabbit anti-Ki67 [SP6] (Abcam, ab16667), rabbit anti-ezrin (Invitrogen, PA5-17518).

### Serum hormone measurements.

Serum total TSH and T_4_ concentrations were measured using radioimmunoassays as previously described (63, 64).

### Thyroid gland size measurement.

Thyroids of euthanized animals were dissected, with both lobes of the gland fully exposed. Images of the neck were captured with a calibrated size marker included in situ. Thyroid gland area (mm^2^, normalized to body weight in grams) was measured using ImageJ (NIH) and quantified as previously described (12).

### Histology and immunostaining of thyroid sections.

Mouse thyroid tissue was immersion-fixed with 10% formalin, paraffin embedded, sectioned, and stained with hematoxylin and eosin. For immunofluorescence, thyroid sections were deparaffinized in Citrisolv, rehydrated using a graded ethanol series, followed by antigen retrieval in citrate buffer, blocking in 1.5 % goat serum, incubation with primary antibodies (overnight, 4°C), followed by 1 hour at room temperature with Alexa Fluor 488– and/or Alexa Fluor 555–conjugated secondary antibodies (Invitrogen and Jackson ImmunoResearch), counterstaining with ProLong Gold and DAPI (Invitrogen), and imaging with Nikon A1 confocal microscope or Leica STELLARIS 8 FALCON confocal microscope. Immunohistochemistry of Ki67 used VECTASTAIN-ABC (Vector Laboratories) with ×40 objective image capture (Olympus EX51 Microscope). Quantitation of Ki67-positive nuclei as a fraction of total thyroid nuclei per field was performed using Imaris software.

### Electron microscopy.

Mouse thyroids were quickly dissected and immersion-fixed in 2.5% glutaraldehyde. The tissue was washed in 100 mM Na cacodylate containing 2 mM CaCl_2_, post-fixed with 0.25 % OsO_4_, washed and then stained with 0.5 % uranyl acetate, washed and dehydrated in a graded ethanol series, incubated 30 minutes in propylene oxide, and infiltrated and polymerized in Araldite. Sections of 70 nm on Formvar-coated copper grids, poststained with 1 % lead citrate, were examined in a JEOL-JEM-1400 transmission electron microscope.

### Western blotting.

Mouse thyroid glands (or PCCL3 cells) were homogenized in RIPA buffer (150 mM NaCl, 25 mM Tris-HCl pH 7.6, 1 % NP-40, 1 % sodium deoxycholate, 0.1 % SDS, Thermo Fisher Scientific) supplemented with protease and phosphatase inhibitor cocktails (Thermo Fisher Scientific), followed by sonication. Protein concentration was measured by BCA assay (Thermo Fisher Scientific). Protein lysates were heated in NuPAGE LDS sample buffer with 50 mM dithiothreitol at 95°C for 5 minutes, resolved by SDS-PAGE, and electrotransferred to nitrocellulose. The membranes were blocked with 5 % milk, immunoblotted with the indicated antibodies and appropriate HRP-conjugated secondary antibody (Bio-Rad, 1721019, 1706516), and visualized by enhanced chemiluminescence. Bands’ intensities were quantified using ImageJ.

### Cell culture and iodination.

PCCL3 cells (from B. DiJeso, University of Salento, Lecce, Italy) and clone 7F cells (35, 56) were cultured in DMEM/F12 (17.5 mM glucose) with 5% bovine calf serum and a 4-hormone (Sigma) mixture containing 1 mIU/mL TSH, 1 μg/mL insulin, 1 nM hydrocortisone, and 5 μg/mL apo-transferrin. For exogenous iodination, PCCL3 cells maintained in complete medium were treated with either an incomplete 2-component mixture (negative controls) or a complete 3-component iodination system that included NaI (100 μM), glucose oxidase (17.8 μg/L ≈ 2.7 U/L, where 1 U generates 1.0 micromole hydrogen peroxide [and D-gluconic acid] per min; Sigma, G7141) and LPO (3 mg/L ≈ 856 U/L, where 1 U oxidizes 1.0 micromole per min; Sigma, L2005). Where indicated in various samples, LPO was omitted; serum was omitted; peroxidase-mediated iodination was blocked with PTU (0.1 mM; Sigma, P3755); and Na^125^I (70 μCi per sample) was added. Independently, a supraphysiological cytotoxic dose of glucose oxidase (442 U/L) and 10-fold excess of PTU (1 mM) were intentionally used in Supplemental Figure 5. Cell death assays are described below.

### Cell death assays.

The ApopTag In Situ Apoptosis Detection Kit (Millipore) was used for fluorescent TUNEL staining of thyroid sections. Sections were counterstained and mounted with ProLong Gold and DAPI. Quantitation was measured as the fraction of follicles bearing TUNEL-positive thyroid cells per 70,000 μm^2^ field (with multiple fields imaged per thyroid gland).

For PCCL3, cells were grown in 8-well chamber slides (Millicell-EZ), washed with PBS, exogenously iodinated (or incubated under control conditions) for 8 hours, fixed with 10% neutral-buffered formalin for 20 minutes at room temperature, and permeabilized (0.1 % Triton X-100 in 0.1% sodium citrate). TUNEL labeling was performed using the in situ cell death detection kit, fluorescein (Roche), counterstained with ProLong Gold and DAPI (Invitrogen), and imaged in a Leica STELLARIS 8 FALCON confocal microscope. TUNEL-positive nuclei as a fraction of DAPI-positive nuclei was quantified using AIVIA Artificial Intelligence-guided software. For each individual biological replicate, 5 random field images were taken and averaged.

For Annexin V and propidium iodide labeling, PCCL3 cells were plated in chambered coverglass and cultured for 2–3 days and then incubated with the exogenous iodination cocktail (or controls) described above for 4 hours. At this time the cells were washed twice with PBS and labeled with Annexin V–FITC and propidium iodide (Sigma) for 10 minutes at room temperature. Multiple random bright-field and corresponding fluorescence images were captured (in a Nikon A1 confocal microscope) for each sample. Total cells under bright-field conditions were quantified using AIVIA Artificial Intelligence-guided software, and from this the Annexin V– and PI-positive fractions were calculated.

For the CytoTox-Glo assay (Promega), PCCL3 cells were seeded in 96-well plates. One day later, the cells were washed twice with PBS and then incubated with the exogenous iodination cocktail (or controls) described above for 24 hours. Cytotoxicity assay was performed by luminescence generated by dead cells, and then by total cells, as measured on a Veritas luminometer (Turner biosystems).

### Statistics.

Comparisons between 2 groups were made by unpaired 2-tailed Student’s *t* test. Comparisons of more than 2 groups were made by 1-way ANOVA with Tukey’s post hoc test. All statistical analyses were conducted with GraphPad Prism. Data are represented as mean ± SD; *P* values of less than 0.05 were considered significant.

### Study approval.

All animal experiments performed with mice were in compliance and approved by the University of Michigan Institutional Animal Care and Use Committee (IACUC, PRO00011324).

### Data availability.

All data (both those shown in the published and supplemental figures as well as replicates not shown and accompanying metadata descriptions) have been uploaded in two master PDF files at the journal’s website and are freely available for download. Additionally, quantitation presented in the figures is documented in a Supporting Data Values file. Reagents are also available upon request (to the corresponding author).

## Author contributions

Designing research studies (CY, XZ, XW, APK, PA). Conducting experiments and acquiring data (CY, XZ, XW, APK, KP, AZC, XHL, EM, HG, DL, HZ). Analyzing data (CY, XZ, XW, APK, DL, EM, PA). Providing reagents (HG, SR). Writing the initial manuscript (PA). Editing the manuscript and approving the final draft (all). CY and XZ contributed equally and their authorship positions assigned by mutual agreement.

## Funding support

This work is the result of NIH funding and is subject to the NIH Public Access Policy. Through acceptance of this federal funding, the NIH has been given a right to make the work publicly available in PubMed Central.

- NIH R01DK132017 to PA, F30DK139717 to CY, and R01DK15070 to SR.
- NIH CA46592 to University of Michigan Cancer Center, for the Transgenic Animal Model Core of the University of Michigan Biomedical Research Core Facilities.
- NIH P30DK020572 and P30EY007003 to University of Michigan Microscopy and Image Analysis Core.
- NIH S10 OD28612-01-A1 for purchase of the Leica STELLARIS 8 FALCON Confocal Microscopy System.

## Supplementary Material

Supplemental data

Unedited blot and gel images

Supporting data values

## Version 1 10/14/2025

In-Press Preview

## Version 2 12/15/2025

Electronic publication

## References

[1] Marciniak SJ, Ron D (2006). Endoplasmic reticulum stress signaling in disease. Physiol Rev.

[2] Rao RV, Bredesen DE (2004). Misfolded proteins, endoplasmic reticulum stress and neurodegeneration. Curr Opin Cell Biol.

[3] Walter P, Ron D (2011). The unfolded protein response: from stress pathway to homeostatic regulation. Science.

[4] Qi L (2017). New insights into the physiological role of endoplasmic reticulum-associated degradation. Trends Cell Biol.

[5] Xu Y, Fang D (2020). Endoplasmic reticulum-associated degradation and beyond: The multitasking roles for HRD1 in immune regulation and autoimmunity. J Autoimmun.

[6] Huang EY (2018). A VCP inhibitor substrate trapping approach (VISTA) enables proteomic profiling of endogenous ERAD substrates. Mol Biol Cell.

[7] Lemmer IL (2021). A guide to understanding endoplasmic reticulum stress in metabolic disorders. Mol Metab.

[8] Shrestha N (2023). Integration of ER protein quality control mechanisms defines β cell function and ER architecture. J Clin Invest.

[9] Haynes CM (2004). Degradation of misfolded proteins prevents ER-derived oxidative stress and cell death. Mol Cell.

[10] McGrath EP (2021). Death sentence: The tale of a fallen endoplasmic reticulum. Biochim Biophys Acta Mol Cell Res.

[11] Hetz C (2020). Mechanisms, regulation and functions of the unfolded protein response. Nat Rev Mol Cell Biol.

[12] Zhang X (2021). Thyroid hormone synthesis continues despite biallelic thyroglobulin mutation with cell death. JCI Insight.

[13] Zhang X (2022). Maintaining the thyroid gland in mutant thyroglobulin-induced hypothyroidism requires thyroid cell proliferation that must continue in adulthood. J Biol Chem.

[14] Zhang X (2022). Defective thyroglobulin: cell biology of disease. Int J Mol Sci.

[15] Holzer G (2016). Thyroglobulin represents a novel molecular architecture of vertebrates. J Biol Chem.

[16] Di Jeso B, Arvan P (2016). Thyroglobulin from molecular and cellular biology to clinical endocrinology. Endocr Rev.

[17] Carvalho DP, Dupuy C (2017). Thyroid hormone biosynthesis and release. Mol Cell Endocrinol.

[18] Dunn JT, Dunn AD (1999). The importance of thyroglobulin structure for thyroid hormone biosynthesis. Biochimie.

[19] Kim K (2021). The structure of natively iodinated bovine thyroglobulin. Acta Crystallogr D Struct Biol.

[20] Coscia F (2020). The structure of human thyroglobulin. Nature.

[21] Marechal N (2022). Formation of thyroid hormone revealed by a cryo-EM structure of native bovine thyroglobulin. Nat Commun.

[22] Adaixo R (2022). Cryo-EM structure of native human thyroglobulin. Nat Commun.

[23] Lamas L (1972). Evidence for a catalytic role for thyroid peroxidase in the conversion of diiodotyrosine to thyroxine. Endocrinology.

[24] Lamas L, Taurog A (1977). The importance of thyroglobulin structure in thyroid peroxidase-catalyzed conversion of diiodotyrosine to thyroxine. Endocrinology.

[26] Di Cosmo C (2010). Mice deficient in MCT8 reveal a mechanism regulating thyroid hormone secretion. J Clin Invest.

[29] Pio MG (2020). A novel mutation in intron 11 donor splice site, responsible of a rare genotype in thyroglobulin gene by altering the pre-mRNA Splicing process. Cell expression and bioinformatic analysis. Mol Cell Endocrinol.

[30] Citterio CE (2019). The role of thyroglobulin in thyroid hormonogenesis. Nat Rev Endocrinol.

[31] Zhang X (2023). Perturbation of endoplasmic reticulum proteostasis triggers tissue injury in the thyroid gland. JCI Insight.

[32] Tong W (1974). Actions of thyroid-stimulating hormone. Handbook of Physiology.

[33] Studer H, Ramelli F (1982). Simple goiter and its variants: euthyroid and hyperthyroid multinodular goiters. Endocr Rev.

[34] Goormaghtigh N, Thomas F (1934). The functional reactions of the human thyroid: a contribution to its histophysiology. Am J Pathol.

[35] Morishita Y (2020). Thyrocyte cell survival and adaptation to chronic endoplasmic reticulum stress due to misfolded thyroglobulin. J Biol Chem.

[36] Kim PS (1996). An endoplasmic reticulum storage disease causing congenital goiter with hypothyroidism. J Cell Biol.

[37] Medeiros-Neto G (1996). Congenital hypothyroid goiter with deficient thyroglobulin. Identification of an endoplasmic reticulum storage disease with induction of molecular chaperones. J Clin Invest.

[38] Morishita Y (2021). Cell death-associated lipid droplet protein CIDE-A is a noncanonical marker of endoplasmic reticulum stress. JCI Insight.

[39] De Deken X, Miot F (2019). DUOX defects and their roles in congenital hypothyroidism. Methods Mol Biol.

[40] Poncelet L (2019). The Dual Oxidase Duox2 stabilized with DuoxA2 in an enzymatic complex at the surface of the cell produces extracellular H_2_O_2_ able to induce DNA damage in an inducible cellular model. Exp Cell Res.

[41] Vitale M (2000). Iodide excess induces apoptosis in thyroid cells through a p53-independent mechanism involving oxidative stress. Endocrinology.

[42] Johnson KR (2007). Congenital hypothyroidism, dwarfism, and hearing impairment caused by a missense mutation in the mouse dual oxidase 2 gene, Duox2. Mol Endocrinol.

[43] Ekholm R, Bjorkman U (1997). Glutathione peroxidase degrades intracellular hydrogen peroxide and thereby inhibits intracellular protein iodination in thyroid epithelium. Endocrinology.

[44] Song Y (2007). Roles of hydrogen peroxide in thyroid physiology and disease. J Clin Endocrinol Metab.

[45] Szanto I (2019). H_2_O_2_ metabolism in normal thyroid cells and in thyroid tumorigenesis: focus on NADPH oxidases. Antioxidants (Basel).

[46] Grieco G (2020). Class III PI3K Vps34 controls thyroid hormone production by regulating thyroglobulin iodination, lysosomal proteolysis, and tissue homeostasis. Thyroid.

[47] Villacorte M (2016). Thyroid follicle development requires Smad1/5- and endothelial cell-dependent basement membrane assembly. Development.

[48] Degosserie J (2018). Extracellular vesicles from endothelial progenitor cells promote thyroid follicle formation. J Extracell Vesicles.

[49] Riou C (1999). Susceptibility of differentiated thyrocytes in primary culture to undergo apoptosis after exposure to hydrogen peroxide: relation with the level of expression of apoptosis regulatory proteins, Bcl-2 and Bax. Endocrinology.

[50] Grasberger H (2012). Mice deficient in dual oxidase maturation factors are severely hypothyroid. Mol Endocrinol.

[51] Taurog A (1976). The mechanism of action of the thioureylene antithyroid drugs. Endocrinology.

[52] Niles AL (2007). A homogeneous assay to measure live and dead cells in the same sample by detecting different protease markers. Anal Biochem.

[53] Zurzolo C (1991). The polarized epithelial phenotype is dominant in hybrids between polarized and unpolarized rat thyroid cell lines. J Cell Sci.

[54] Taurog A (1974). Comparison of lactoperoxidase- and thyroid peroxidase-catalyzed iodination and coupling. Endocrinology.

[55] Roy G, Mugesh G (2005). Anti-thyroid drugs and thyroid hormone synthesis: effect of methimazole derivatives on peroxidase-catalyzed reactions. J Am Chem Soc.

[56] Citterio CE (2018). Relationship between the dimerization of thyroglobulin and its ability to form triiodothyronine. J Biol Chem.

[57] Barrett KL (2001). Advances in cytochemical methods for detection of apoptosis. J Histochem Cytochem.

[58] Gonzalez-Guerrero C (2023). Iodotyrosines are biomarkers for preclinical stages of iodine-deficient hypothyroidism in *Dehal1*-knockout mice. Thyroid.

[59] Sano R, Reed JC (2013). ER stress-induced cell death mechanisms. Biochim Biophys Acta.

[60] Iurlaro R, Munoz-Pinedo C (2016). Cell death induced by endoplasmic reticulum stress. FEBS J.

[61] Kohrle J (2023). Selenium, iodine and iron-essential trace elements for thyroid hormone synthesis and metabolism. Int J Mol Sci.

[62] Coxon KM (2011). Purification of annexin V and its use in the detection of apoptotic cells. Methods Mol Biol.

[63] Ferrara AM (2013). Changes in thyroid status during perinatal development of MCT8-deficient male mice. Endocrinology.

[64] Pohlenz J (1999). Improved radioimmunoassay for measurement of mouse thyrotropin in serum: strain differences in thyrotropin concentration and thyrotroph sensitivity to thyroid hormone. Thyroid.

