# Supplementary material for: Stimulated thyroid hormone synthesis machinery drives thyrocyte cell death independent of ER stress
Source: J Clin Invest. 2025 Oct 14;135(24):e187044. doi: 10.1172/JCI187044 (PMC12700552; doi:10.1172/JCI187044)
Supplement: Supplemental data [file jci-135-187044-s142.pdf]

Supplement

Supplemental Figure Legends

Figure S1. **Generation of *Tg-KO* mice**. Homologous recombination was performed in mouse ES cells and clones selected and screened (see Methods).

A) Plasmids used for design of recombination fragment.

B) Diagram drawn to scale of a portion endogenous mouse *Tg* (upper line), the regions of identity (lighter green segments, middle line), and the targeted region (red, middle line) replaced by neo cassette (dark green, lower line) upon homologous recombination.

C) Initial detection of positive ES clone by conventional PCR.

D) Southern blotting of DNA digest from ES cells.

E) Genotyping of tail-biopsy genomic DNA.

F) Reverse-transcribed RNA from thyroid tissue of WT and *Tg*-KO mice was analyzed by qPCR using primer pairs designed to probe various regions along the length of the *Tg* mRNA shown in the cartoon above (and see Methods) – greater than 40 cycles was read as zero.

G) Western blotting of TG from thyroid tissue isolated from the genotypes noted above (*below, actin is a loading control*).

H) Neck dissection revealing thyroid glands of WT and *Tg*-KO mice (orange arrows highlight goitrous left and right lobes), age 4.5 months.

Figure S2. **Thyroxine withdrawal from *Tg*-KO mice**. *Tg*-KO pups suckled from thyroxine treated mothers were moved to a fresh cage upon weaning with thyroxine supplementation in the drinking water. At the time of initial withdrawal of thyroxine supplementation and at the indicated times thereafter, animals were euthanized, and fixed thyroid gland tissue sectioned, stained (H&E), and imaged at a fixed magnification. A 200 µm size marker is indicated.

Figure S3. **In *Tg*-KO mice, cells entrapped within the thyroid follicular lumen are thyrocytes, despite the absence of ER stress.**

A) PAX8 immunofluorescence (*red*, with DAPI blue counterstain) in *Tg*-KO mouse thyroids (n=2-3 per group, 1 mo). Yellow boxes denote follicles with PAX8-positive nuclei within the thyroid follicle lumen.

B) Immunofluorescence of BiP staining (*green*, with DAPI blue counterstain) in WT C57BL6/j and *Tg*-KO mice (n=3-4 per group, 1 mo). Scale bars are indicated.

Figure S4. ***$Tg^{+/-}$* mice are protected from the cell death exhibited by *Tg*-KO mice have.**

A) Cleaved Caspase-3 (by Western blotting) in the thyroid glands of WT C57BL6/j and *Tg*-KO mice (n=2-4 per group; each lane represents a different animal).

B) *Left:* TUNEL labeling (*red*, with DAPI blue counterstain) in thyroid sections of $Tg^{+/+}$ and $Tg^{+/-}$ heterozygous mice (n=2-5 per group, age 6.5 ± 2.9 mo; scale bar=50 μm).
Right: Quantitation of the percentage of follicles containing TUNEL-positive cells in proportion to total follicles in each thyroid section image from $Tg^{+/-}$ heterozygous mice and *Tg*-KO mice (re-shown from Fig. 4B, for purposes of comparison). Each color represents a different animal; each point is an independent image; males=squares, females=circles; mean ± SD; ***p<0.001 (unpaired 2-tailed Student's *t* test).

Figure S5. Thyroid cell death in *Tg*-KO mice is inducible upon T_4 withdrawal. A) *Tg*-KO mice treated with T_4 were subjected to T_4 withdrawal for the indicated periods number of days (n number of animals in each group shown at bottom). All animals were euthanized at the same final age; random thyroid sections were taken for TUNEL staining (4 sections per each animal). TUNEL images from each cohort of animals were arranged from least-to-greatest thyroid cell death and the median image was selected for presentation in panel A. **B)** Serum TSH measurement of *Tg*-KO mice treated with T4 and subjected to T4 withdrawal for the indicated number of days. **C)** Quantitation of all thyroid TUNEL staining images (a different color was chosen for each animal; squares = males, circles = females; ****p<0.0001, 1-way ANOVA with Tukey's post hoc test).

Figure S6. PCCL3 thyrocytes can be killed with supraphysiological H_2O_2 generation. PCCL3 cells (complete medium containing 17.5 mM glucose) were treated with glucose oxidase at either 2.7 U/L or more than 160-fold greater dose (442 U/L). The CytoTox-Glo cytotoxicity assay was measured at 24 hours. Cytotoxicity at a supraphysiological levels of H_2O_2 generation cannot be inhibited even at a dose of 1 mM propylthiouracil (PTU).

Figure S7. Peroxidase-mediated cytotoxicity is concurrent with protein iodination. Clone 7F (*Tg*-KO PCCL3) cells were rinsed once in PBS, re-fed complete medium lacking serum, and treated for 4 h in the presence of the listed components at the identical concentrations to those described in Figure 6. For the samples in lanes 1, 2, 4, and 5, $Na^{125}I$ (70 μCi per sample) was added. To detect the presence of iodoproteins, cell lysates were analyzed by SDS-PAGE and phosphorimaging. The samples in lane 3 comes from cells incubated in the complete absence of tracer (i.e., without radioiodide); this lane is separated from neighboring lanes by white lines but all lanes come from the same gel and exposure). The positions of molecular weight standards are indicated.

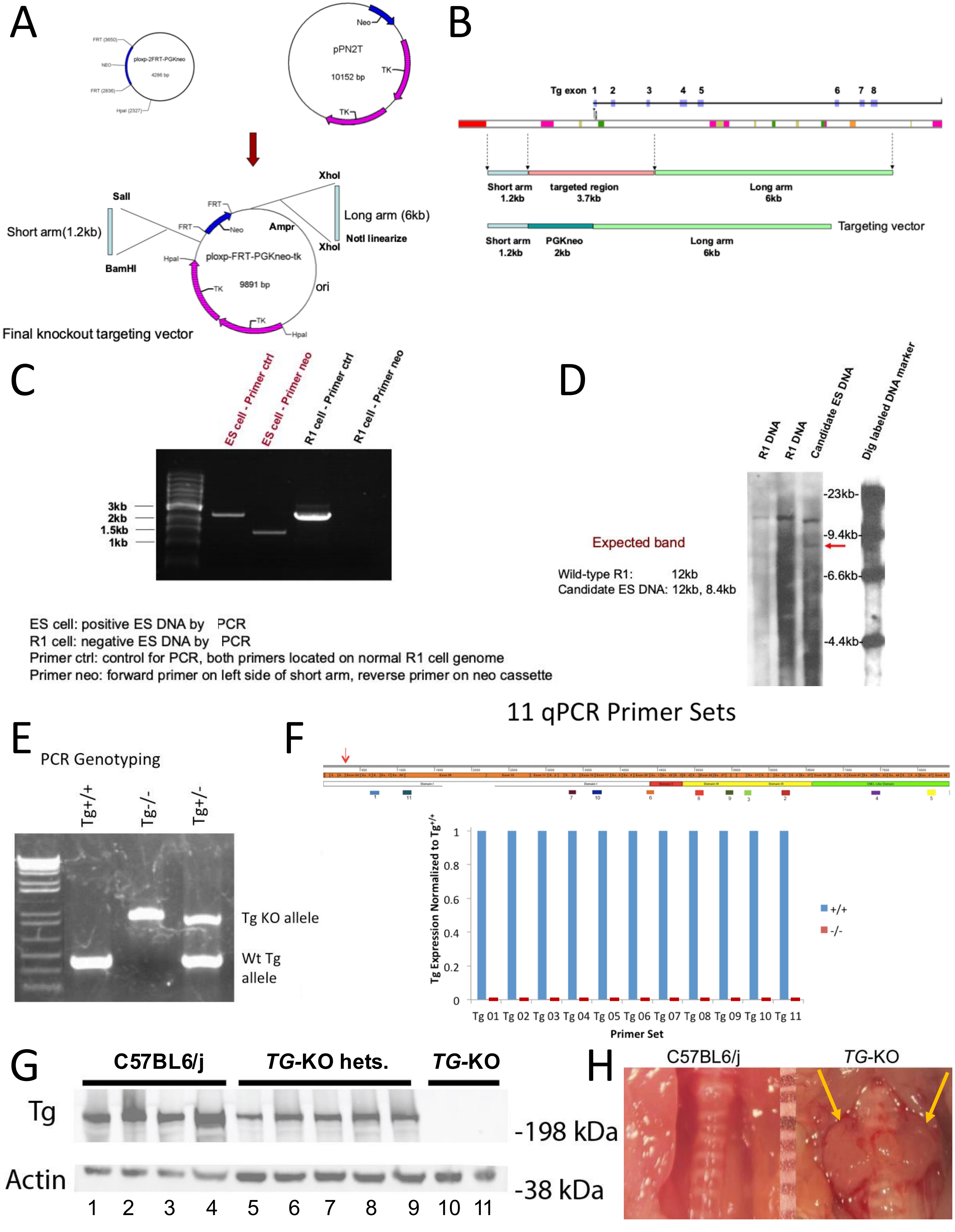

Young, Zhang, Supplemental Fig. S1

On T_4 Supplementation

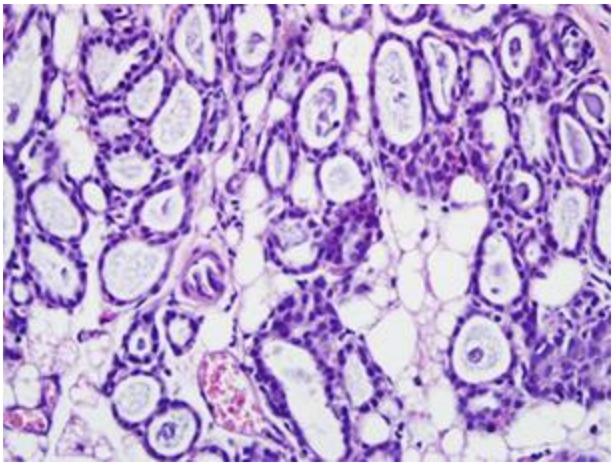

T_4 Withdrawal (1 week)

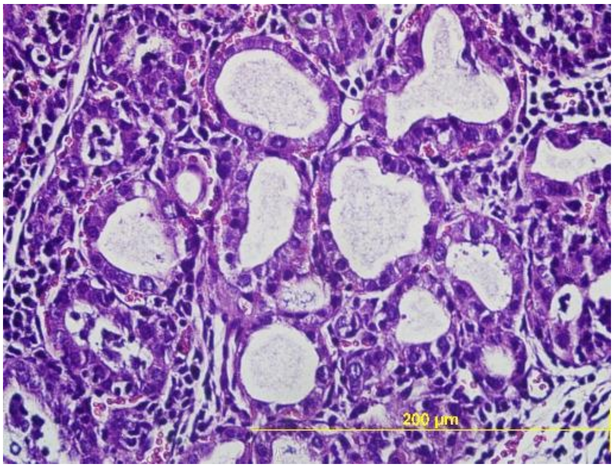

T_4 Withdrawal (2 week)

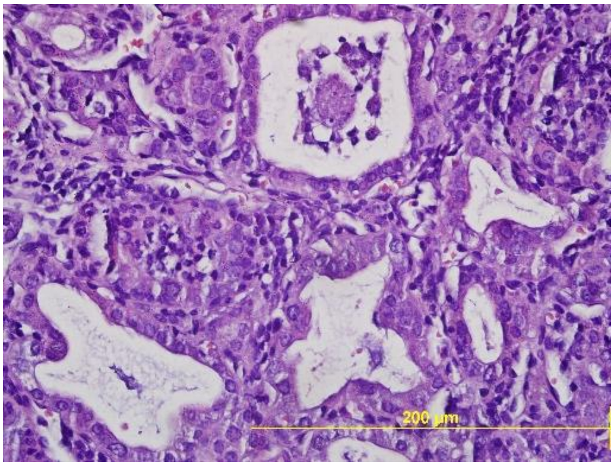

T_4 Withdrawal (4 week)

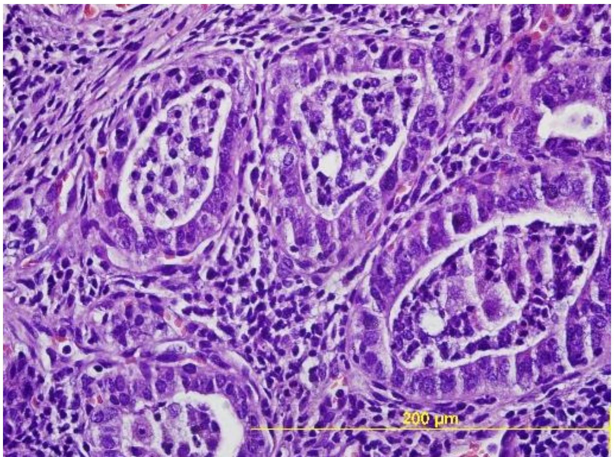

Young, Zhang, Supplemental Fig. S2

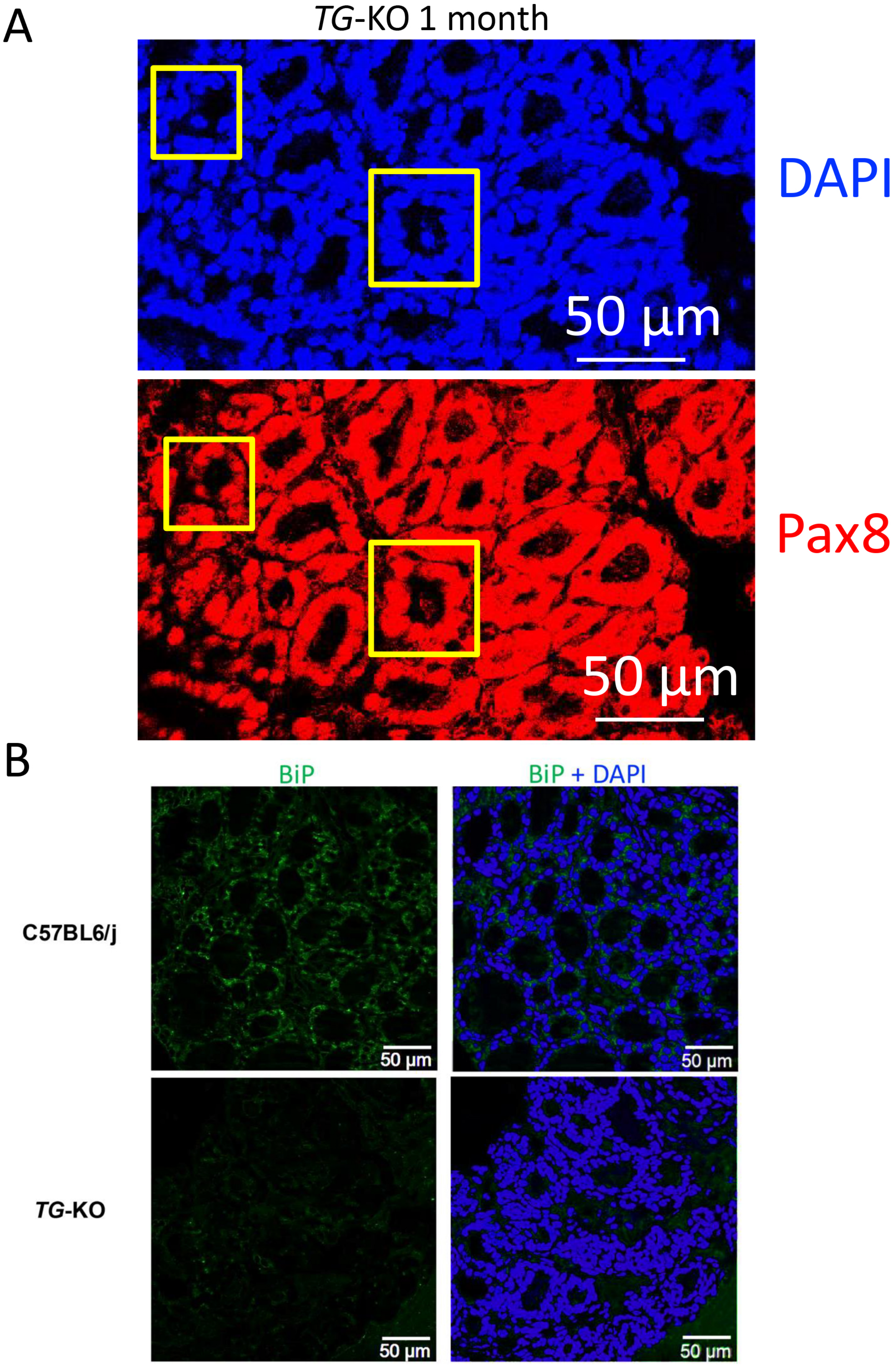

Young, Zhang, Supplemental Fig. S3

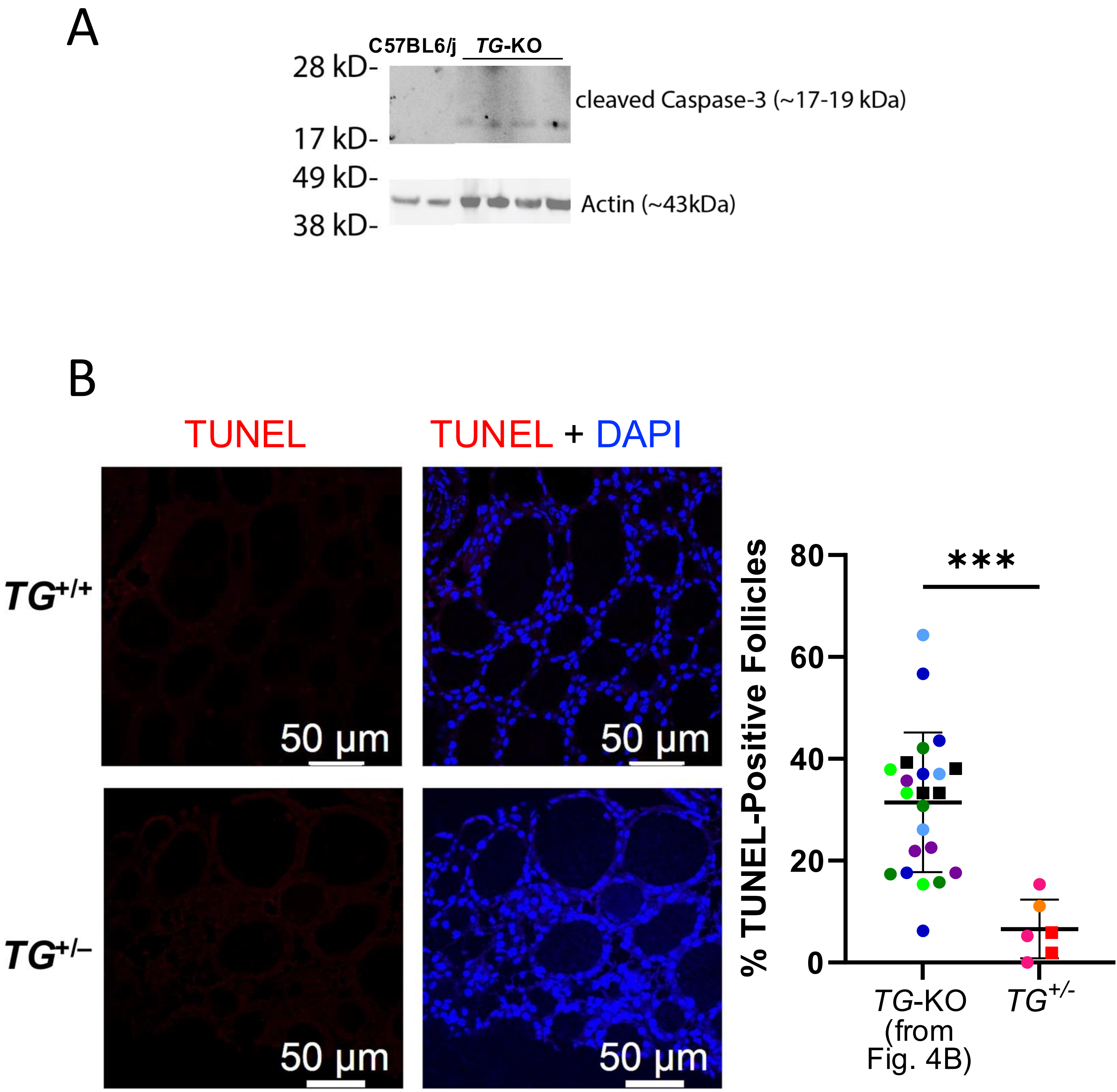
A
C57BL6/j
TG-KO
28 kD-
17 kD-
cleaved Caspase-3 (~17-19 kDa)
49 kD-
38 kD-
Actin (~43kDa)
B
TUNEL
TUNEL + DAPI
TG+/+
TG+/–
50 µm
% TUNEL-Positive Follicles
***
TG-KO (from Fig. 4B)
TG+/-

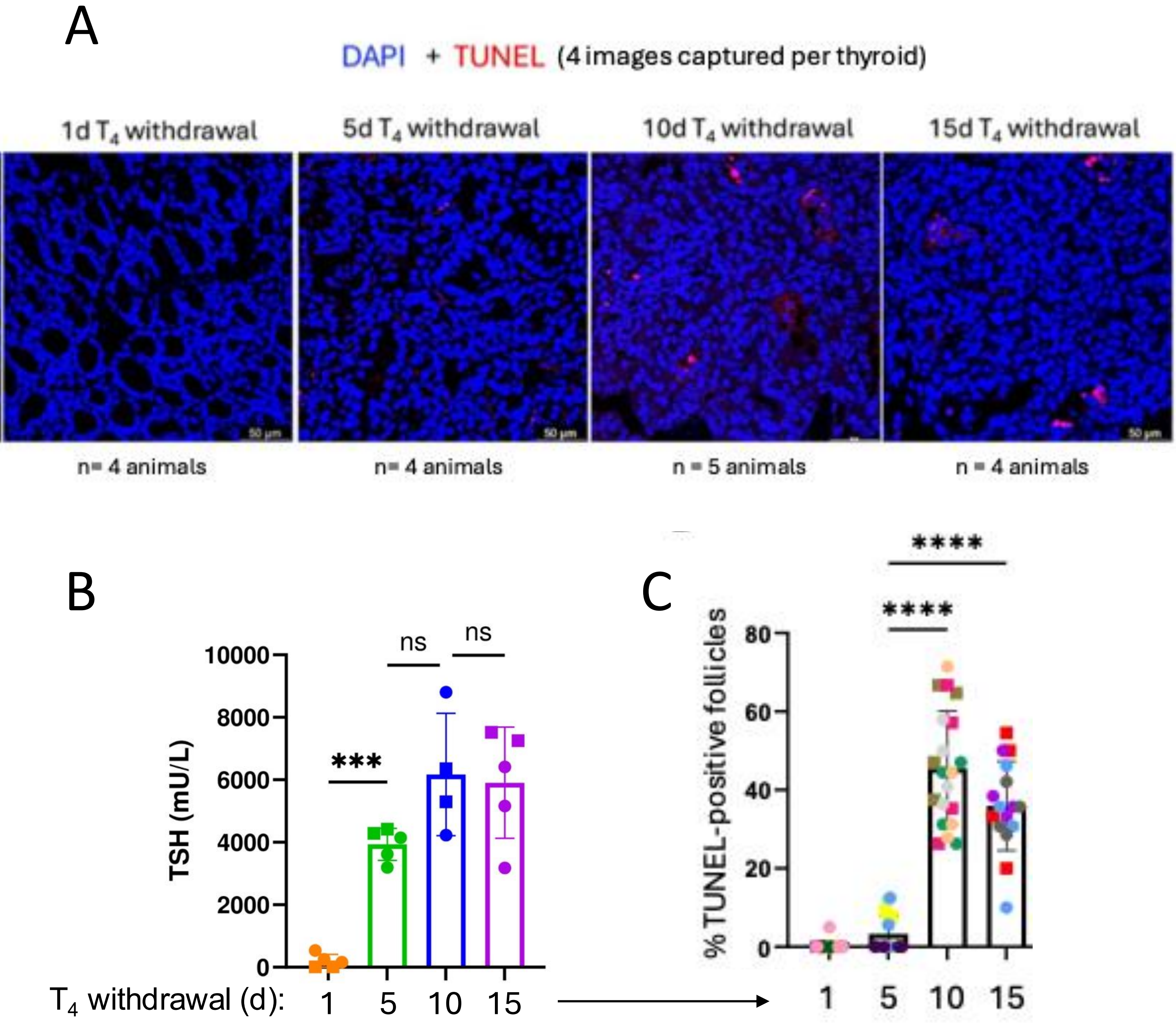

Young, Zhang, Supplemental Fig. S5

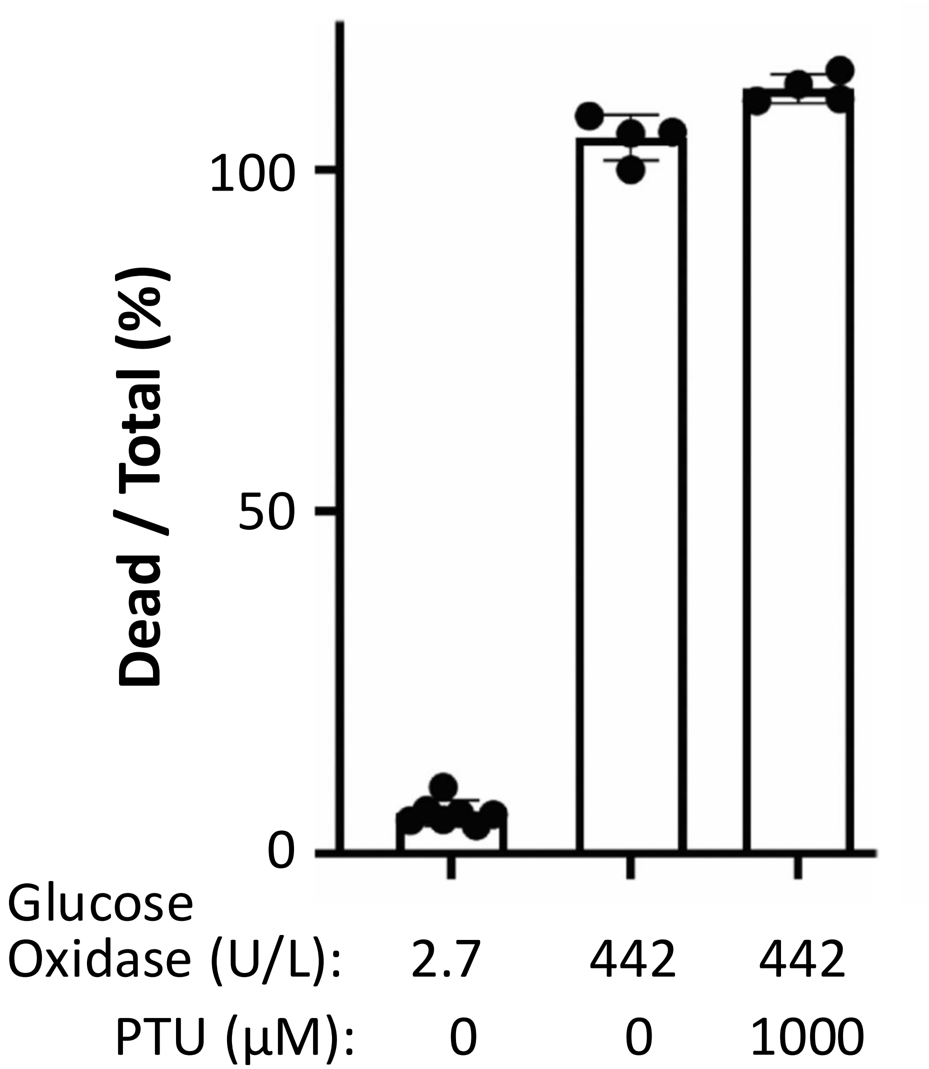

Dead / Total (%)
100
50
0
Glucose
Oxidase (U/L): 2.7 442 442
PTU (µM): 0 0 1000

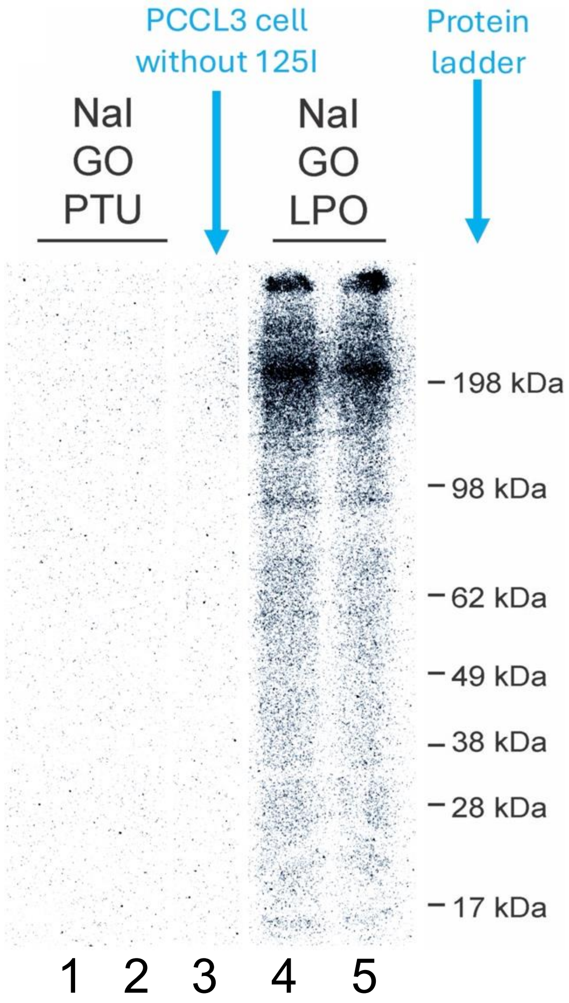

Young, Zhang, Supplemental Fig. S7
