# Supplementary material for: Stimulated thyroid hormone synthesis machinery drives thyrocyte cell death independent of ER stress
Source: J Clin Invest. 2025 Oct 14;135(24):e187044. doi: 10.1172/JCI187044 (PMC12700552; doi:10.1172/JCI187044)

Fig 2B

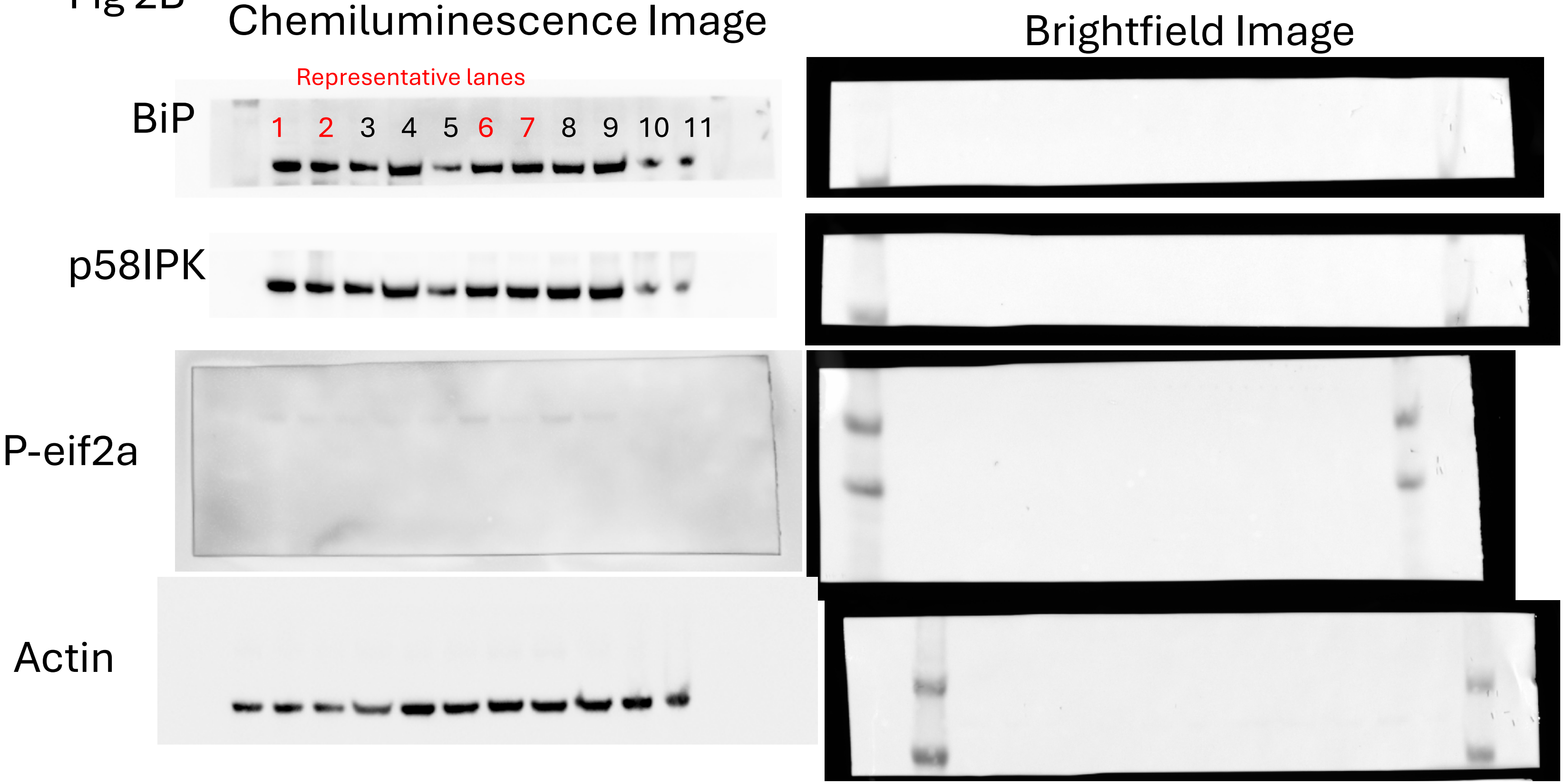

Lane	Genotype	mouse #	Age
1	B6	18661	1.4 mo
2	B6	18659	1.4 mo
3	B6	18658	1.4 mo
4	B6	18469	2.9 mo
5	$Tg^{+/-}$	18128	2.8 mo
6	$Tg^{+/-}$	18129	2.8 mo
7	$Tg^{+/-}$	18130	2.8 mo
8	$Tg^{+/-}$	18134	2.8 mo
9	$Tg^{+/-}$	18135	2.8 mo
10	$Tg^{-/-}$	18245	1 mo
11	$Tg^{-/-}$	18244	1 mo

Fig 2B

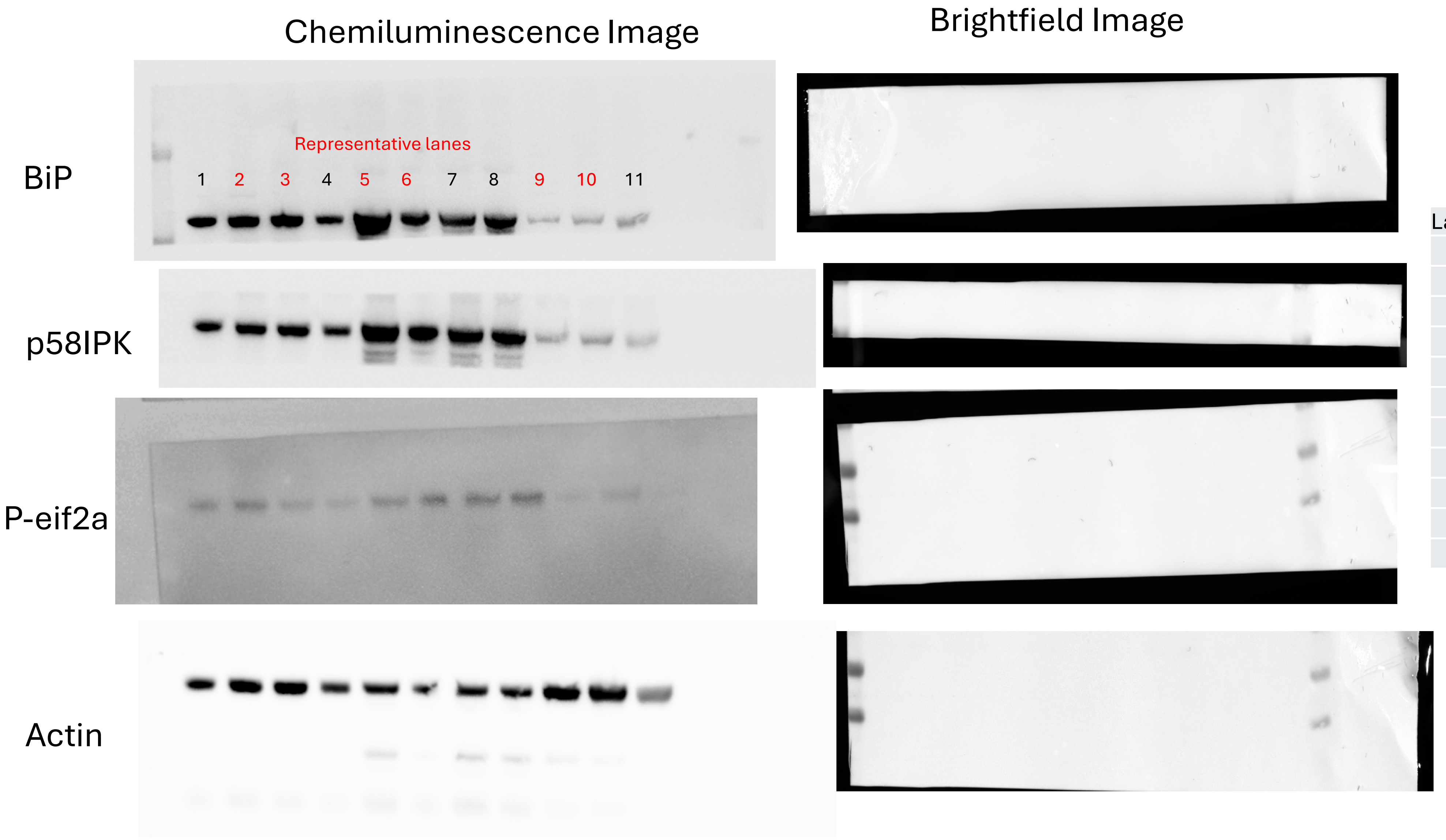

Lane	Genotype	mouse #	Age
1	B6	18661	1.4 mo
2	B6	18659	1.4 mo
3	B6	18658	1.4 mo
4	B6	18423	4.4 mo
5	*cog/cog*	17944	2.6 mo
6	*cog/cog*	17948	2.6 mo
7	*cog/cog*	17949	2.6 mo
8	*cog/cog*	17950	2.6 mo
9	*Tg*-KO	18240	1 mo
10	*Tg*-KO	18244	1 mo
11	*Tg*-KO	18436	1 mo

Fig S1C

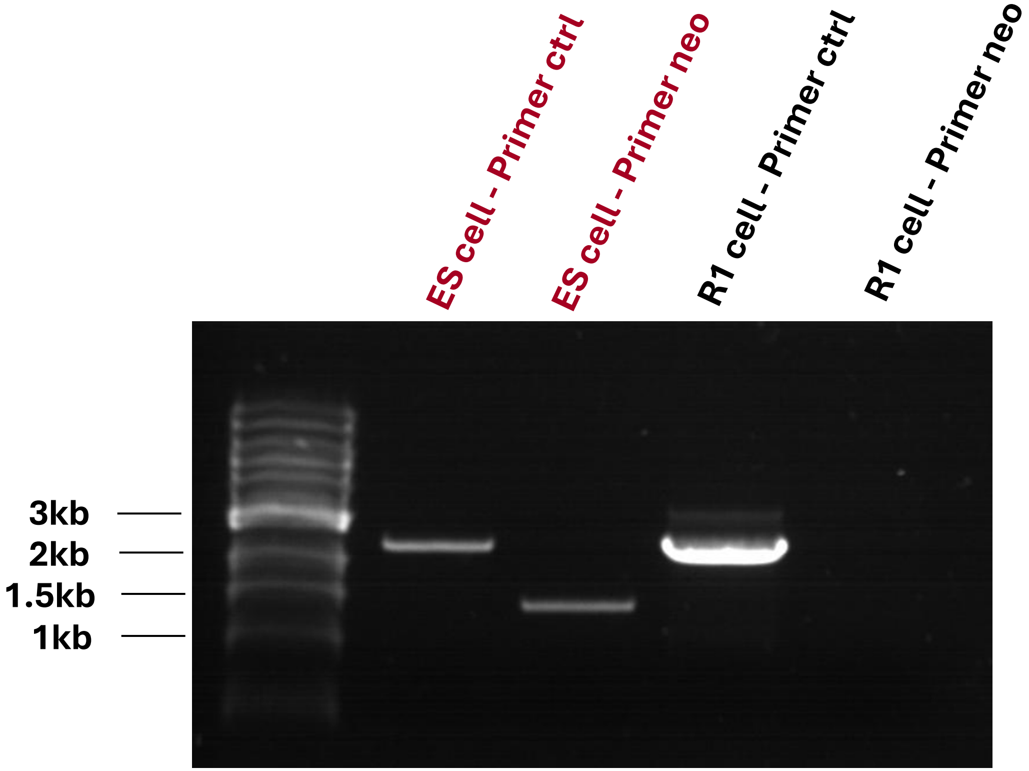

ES cell: positive ES DNA by qPCR
R1 cell: negative ES DNA by qPCR
Primer ctrl: control for PCR, both primers located on normal R1 cell genome
Primer neo: forward primer on left side of short arm, reverse primer on neo cassette

Fig S1D

Expected band

Wild-type R1: 12kb
Candidate ES DNA: 12kb, 8.4kb

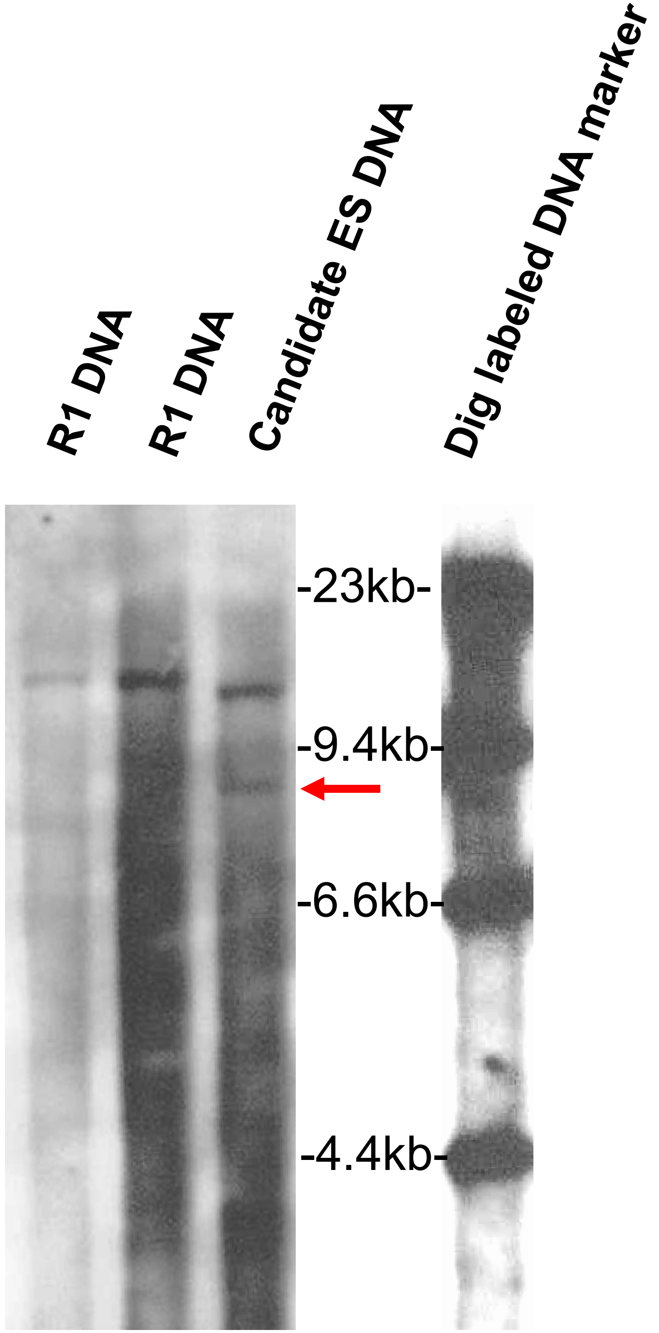

Fig S1G

Chemiluminescence Image

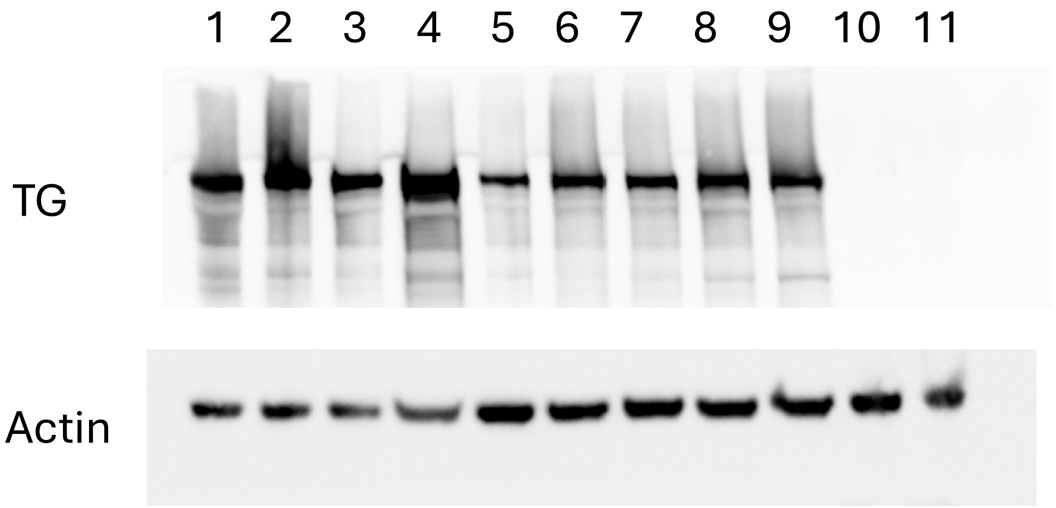

Brightfield Image

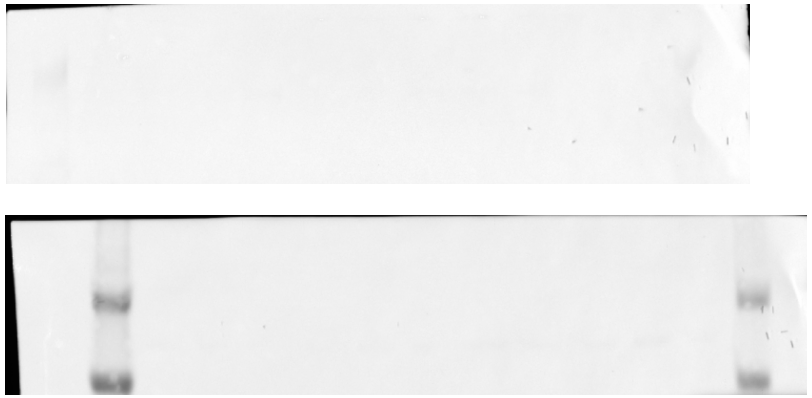

Lane	Genotype	mouse #	Age
1	B6	18661	1.4 mo
2	B6	18659	1.4 mo
3	B6	18658	1.4 mo
4	B6	18469	2.9 mo
5	*Tg*$^{+/-}$	18128	2.8 mo
6	*Tg*$^{+/-}$	18129	2.8 mo
7	*Tg*$^{+/-}$	18130	2.8 mo
8	*Tg*$^{+/-}$	18134	2.8 mo
9	*Tg*$^{+/-}$	18135	2.8 mo
10	*Tg*$^{-/-}$	18245	1 mo
11	*Tg*$^{-/-}$	18244	1 mo

Fig S4A

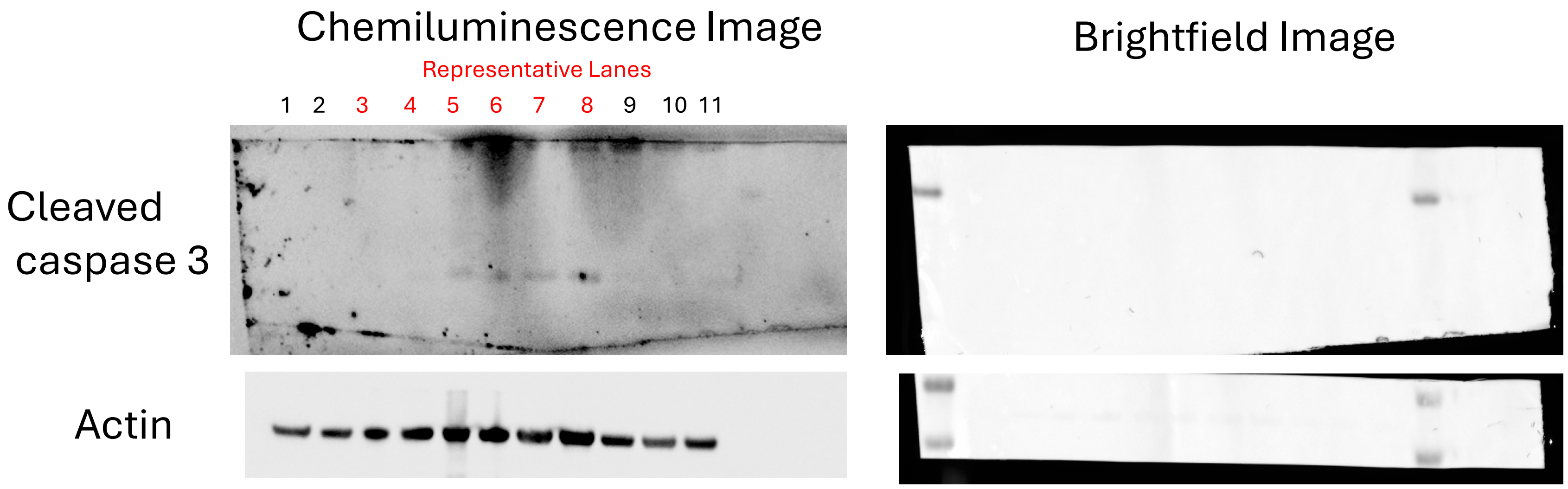

Lane	Genotype	mouse #	Age
1	$Tg^{cog/cog}$	17863	2.9 mo
2	$Tg^{cog/cog}$	17947	2.6 mo
3	B6	18469	2.9 mo
4	B6	18468	2.9 mo
5	$Tg^{-/-}$	18330	3 mo
6	$Tg^{-/-}$	18331	3 mo
7	$Tg^{-/-}$	18241	1 mo
8	$Tg^{-/-}$	18242	1 mo
9	HEK 293	N/A	N/A
10	HEK 293 + 200 ng/mL tunicamycin	N/A	N/A
11	HEK 293 + 500 ng/mL tunicamycin	N/A	N/A

Fig S7

Lane 1, 2: NaI GO PTU
Lane3: pccl3 cells without 125I
Lane 4, 5: NaI GO LPO

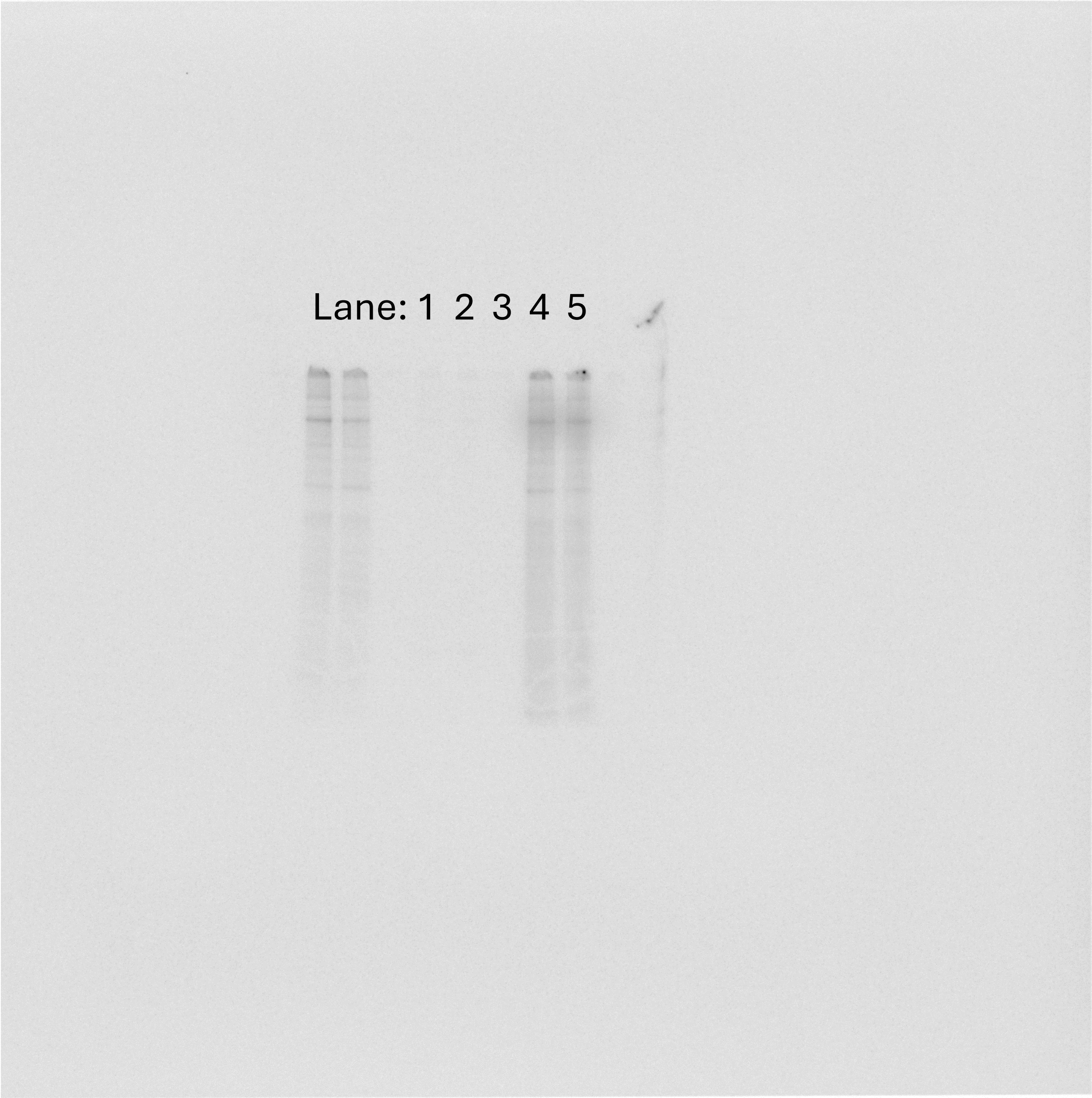

Supplement: Unedited blot and gel images [file jci-135-187044-s143.pdf]
